# Integrating copy number data of 64 iAMP21 BCP-ALL patients narrows the common region of amplification to 1.57 Mb

**DOI:** 10.3389/fonc.2023.1128560

**Published:** 2023-02-23

**Authors:** Femke M. Hormann, Alex Q. Hoogkamer, Aurélie Boeree, Edwin Sonneveld, Gabriele Escherich, Monique L. den Boer, Judith M. Boer

**Affiliations:** ^1^ Princess Máxima Center for Pediatric Oncology, Utrecht, Netherlands; ^2^ Oncode Institute, Utrecht, Netherlands; ^3^ Erasmus Medical Center (MC) - Sophia Children’s Hospital, Department of Pediatric Oncology and Hematology, Rotterdam, Netherlands; ^4^ Dutch Childhood Oncology Group, Utrecht, Netherlands; ^5^ Cooperative study group for childhood acute lymphoblastic leukaemia (COALL) – German Cooperative Study Group for Childhood Acute Lymphoblastic Leukemia, Hamburg, Germany; ^6^ Clinic of Pediatric Hematology and Oncology, University Medical Center Hamburg-Eppendorf, Hamburg, Germany

**Keywords:** intrachromosomal amplification of chromosome 21 (iAMP21), peadiatric, acute lymphoblastic leukaemia, genomics, RIPPLY3

## Abstract

**Background and purpose:**

Intrachromosomal amplification of chromosome 21 (iAMP21) is a rare subtype of B-cell precursor acute lymphoblastic leukaemia (BCP-ALL). It is unknown how iAMP21 contributes to leukaemia. The currently known commonly amplified region is 5.1 Mb.

**Methods:**

We aimed to narrow down the common region of amplification by using high resolution techniques. Array comparative genomic hybridization (aCGH) was used to determine copy number aberrations, Affymetrix U133 Plus2 expression arrays were used to determine gene expression. Genome-wide expression correlations were evaluated using Globaltest.

**Results:**

We narrowed down the common region of amplification by combining copy number data from 12 iAMP21 cases with 52 cases from literature. The combined common region of amplification was 1.57 Mb, located from 36.07 to 37.64 Mb (GRCh38). This region is located telomeric from, but not including, *RUNX1*, which is the locus commonly used to diagnose iAMP21. This narrow region, which falls inside the Down Syndrome critical region, includes 13 genes of which the expression of eight genes was significantly upregulated compared with 143 non-iAMP21 B-other cases. Among these, transcriptional repressor *RIPPLY3* (also known as *DSCR6*) was the highest overexpressed gene (fold change = 4.2, FDR < 0.001) and most strongly correlated (R = 0.58) with iAMP21-related genome-wide expression changes.

**Discussion:**

The more precise definition of the common region of amplification could be beneficial in the diagnosis of iAMP21 based on copy number analysis from DNA sequencing or arrays as well as stimulate functional research into the role of the included genes in iAMP21 biology.

## Introduction

Paediatric B-cell acute lymphoblastic leukaemia (BCP-ALL) is the most common childhood malignancy. BCP-ALL consists of several subtypes, each one defined by different chromosomal rearrangements, associated with different prognoses. One of these rearrangements is an intrachromosomal amplification of chromosome 21 (iAMP21). The iAMP21 subtype was discovered in 2003 during routine fluorescence *in situ* hybridization (FISH) screening for the *ETV6-RUNX1* fusion gene, when amplifications of the *RUNX1* gene were observed (1, 2). Currently, the most commonly used definition of iAMP21 is *”three or more extra copies of RUNX1 on a single abnormal chromosome 21 (a total of five or more RUNX1 signals per cell)*” (3, 4). Recent studies have focussed more on the distinctive genomic profile of chromosome 21 (5, 6). iAMP21 BCP-ALL accounts for ~2% of all BCP-ALL cases and often occurs in older children presenting with a low white blood cell count at diagnosis (7). The prognosis of this subtype is poor when treated on a standard protocol (8, 9), but the prognosis improves with more intensive treatment (7, 10). Although some iAMP21 cases harbour concomitant lesions that might be targetable (11, 12), no targeted treatment is available for this high risk subtype.

iAMP21 is characterized by an abnormal chromosome 21, showing structural aberrations such as amplifications, losses, inversions, and other rearrangements. This chromosome is thought to arise through a breakage-fusion-bridge mechanism, often followed by chromothripsis (4, 13–15). While the iAMP21 arises through chromosomal instability, the final chromosome remains stable (4, 16). As can be expected from the formation process, the formed iAMP21 is unique for each case (4, 15). However, Rand et al. (4) show that the amplification is not completely random, with a region that is amplified in 18 studied cases of 5.1 Mb spanning from 32.8 to 37.9 Mb (hg18). This amplified region contains 86 genes (coding and non-coding) (4), and even though some of these genes are highly overexpressed in iAMP21 (17), none of these genes have yet been identified to be causally deregulated by the amplification. Besides overexpression of genes on chromosome 21 (cis-effect), many genes outside of chromosome 21 are overexpressed as well (17), opening up the possibility of an indirect leukemic effect of chromosome 21 amplification (trans-effect).

We aimed to narrow down the common region of amplification (CRA) by combining our patients’ data with published data sets and to pinpoint pivotal genes by integrating analysis of copy number and gene expression data. Our results suggest possible targets of the transformation involved in iAMP21. In addition, narrowing down the CRA in iAMP21 may help the diagnosis of this high-risk subtype based on copy number profiles.

## Methods

### Patient cohort

Bone marrow and/or peripheral blood samples were collected from children with BCP-ALL at time of diagnosis. In accordance with the declaration of Helsinki, and as approved by the Medical Ethics Committee of the Erasmus Medical Center, Rotterdam, The Netherlands, written informed consent to use excess diagnostic material for research purposes was obtained from parents or guardians. Children with newly diagnosed ALL in three consecutive Dutch Childhood Oncology Group trials (DCOG ALL-8, ALL-9, and ALL-10) (18), and two German Cooperative ALL trials (COALL 06-97 and 07-03) (19) were included in this study. The major known cytogenetic subtypes of BCP-ALL, *BCR*-*ABL1*, *ETV6*-*RUNX1*, *KMT2A*/*MLL*-rearranged, *TCF3*-*PBX1*, as well as ploidy status (high hyperdiploidy; 51-65 chromosomes, near tetraploidy; >65 chromosomes, and low hypodiploidy; <39 chromosomes) were determined using karyotyping, fluorescence *in situ* hybridization (FISH) and RT-PCR by reference laboratories. Mononuclear cells were obtained from bone marrow and/or peripheral blood samples by Lymphoprep density gradient centrifugation and blast percentage was determined based on morphology using May-Grunwald-Giemsa staining (20). If necessary, samples were enriched to >90% leukemic blasts using negative beads enrichment. RNA and DNA were routinely isolated from samples using TRIzol reagents.

### Copy number profiles

Copy number was determined in a population-based paediatric ALL cohort (n=372) using the Agilent G3 Human 4x180K aCGH (reference genome build hg18), co-hybridized with 1 µg patient DNA (ULS-Cy5-labeled) and 1 µg reference genomic DNA male pool (ULS-Cy3-labeled), as previously described (21). Agilent Feature Extraction software was used to generate normalized log Ratios. CGHcall was used to normalize the data. The data was dewaved using the NoWave package (22) and 16 references samples, excluding the Y chromosome. Data was centralized using the CGHnormaliter package (23). CGHcall was used to generate segmented data. Segmented LogR values (log2) were used to determine the CRA. Normalized LogR values (log2) were used for the correlation between copy number and gene expression. Copy number data are available at GEO under accession number GSE184692 (see **Supplementary Table S1** for used samples). Supplementary SNP array data (reference genome build hg19) from Gu et al. (24) were used to extend the number of samples. All iAMP21 cases with available SNP array data were selected, excluding SJBALL042258, which is a 60-year-old patient, and SJBALL014273, which showed no apparent iAMP21 copy number profile. Borders of the CRA in hg18 builds were converted to hg19 using the UCSC liftover tool.

### Gene expression

Gene expression microarrays (Affymetrix U133 Plus 2) from a previously described population-based paediatric ALL cohort were used (25, 26). In short, expression data was normalized using vsnrma (27), and batch effects were removed using the empirical Bayes method (28). Differential gene expression between 12 iAMP21 and 143 B-other cases was determined using Limma with false discovery rate (FDR) multiple testing correction (29). Gene expression data are available at GEO under accession number GSE87070 (see **Supplementary Table S1** for used samples). As a validation cohort, RNA sequencing gene expression data from the Pediatric Cancer (PeCan) database (https://pecan.stjude.cloud/) were used. Gene expression in fragments per kilobase per million mapped reads (FPKM) of selected genes was extracted. BCP-ALL samples annotated as *BCR-ABL1*, *ETV6-RUNX1*, *TCF3-PBX1*, high hyperdiploidy, low hypodiploidy, and infant ALL were excluded from analysis, resulting in a validation cohort of 17 iAMP21 samples and 174 B-other samples. Differential gene expression of target genes was determined using the Mann Whitney U test with Bonferroni multiple testing correction.

### Data integration

To determine the correlation between copy number and gene expression one copy number probe and one gene expression probeset per gene were selected. Selection was based on the best correlation between copy number and gene expression (spearman correlation, determined by p-value) and most significant overexpression in iAMP21 compared with B-other cases. Genome-wide gene expression integration, or gene-set testing, was done using the Globaltest package (30), using gene expression of the selected probeset as the response variable and genome-wide gene expression as the covariate. The Globaltest tests the correlation of the input probeset to all other gene expression probesets as a set (global p-value, Bonferroni multiple testing corrected) and to all other gene expression probesets individually (local p-value, FDR multiple testing corrected within each gene-set, correlation signature).

### Statistical approach

Bonferroni corrected p-values were considered significant if <0.05, FDR corrected p-values were considered significant if <0.01. Boxplots show the median (thick line), first and third quartile (box), and minimum and maximum (whiskers; to the furthest distance of 1.5 times the interquartile range). R (version 3.6.3) and Rstudio were used for all data analyses.

## Results

### Patient characteristics

We selected 155 BCP-ALL samples without major known cytogenetic subtypes for which both gene expression array and aCGH data was available (**Supplementary Figure S1**). Among these 155 samples (**Table 1**) 12 cases showed a copy number profile suggestive of iAMP21 (**Supplementary Figure S2**). Of the 143 remaining cases, collectively called B-other, 16 samples had a gain of chromosome 21 (of which 6 Down syndrome (DS) cases) and 4 samples had another structural aberration on chromosome 21 (osa21; data not shown). iAMP21 cases had a lower white blood cell count (median 7.6 *vs* 30 x 10^9^/L, p=0.0056 (Mann Whitney-U)) than the remaining B-other cases. Similarly to previous reports (7), *RB1* deletion (p <0.001) and *EBF1* deletion (p = 0.043) were more frequent, while *PAX5* deletions (p = 0.0035) and *CDKN2A* deletions (p = 0.0061) were less frequent in the iAMP21 group (**Table 1**). Clinical characteristics and treatment outcome of the 12 individual iAMP21 patients are detailed in **Supplementary Table S2**. Interestingly, based on karyotypes, we found one iAMP21 DS patient (iAMP21-06) and one non-DS patient (iAMP21-10), both with an apparent ring chromosome 21 based on karyotype, which is not often described (**Supplementary Table S2**) (7). As only aberrant karyotypes were available, it was not possible to determine whether the ring chromosome is constitutional or acquired in the leukemic cells.

**Table 1:** Clinical characteristics of included patients.

	iAMP21		B-other		
	No. of cases (n=12)	%	No. of cases (n=143)	%	p-value (Fisher exact, Mann-Whitney U)
Gender					
female	5	41.7	64	44.8	1
male	7	58.3	79	55.2	
Age (years)					
median (IQR)	8.5 (6.8-12)		6.0 (2.5-11.5)		0.13
<10	7	58.3	95	66.4	0.55
≧ 10	5	41.7	48	33.6	
WBC count (10^9^/L)					
median (IQR)	7.6 (3.6-16.1)		30 (10.2-91.1)		0.0056
<50	10	83.3	83	58.0	0.13
≧50	2	16.7	60	42.0	
Subtype^1^					
B-other	1	8.3	84	58.7	0.0013
*BCR-ABL1*-like	11	91.7	59	41.3	
MRD at end-of-induction					
Low (<0.0001)	4	40.0	40	53.3	0.51
High (≥0.0001)	6	60.0	35	46.7	
not performed	2		68		
Risk stratification					
Non-HR	6	50.0	68/140	48.6	1
HR	6	50.0	72/140	51.4	
Deletions in B-cell development genes^2^					
*BTG1*	0	0.0	10/141	7.1	1
*IKZF1*	4	33.3	50/140	35.7	1
*EBF1*	3	25.0	8/140	5.7	0.043
*ETV6*	2	16.7	31/142	21.8	1
*PAX5*	0	0.0	59/141	41.8	0.0035
*RB1*	7	58.3	8/141	5.7	<0.001
*CDKN2A*	1	8.3	70/142	49.3	0.0061
*CDKN2B*	3	25.0	68/141	48.2	0.14
*PAR1*	2	16.7	16/142	11.3	0.63

^1^As previously determined by den Boer et al. (31), iAMP21 cases are enriched in the BCR-ABL1-like group (32).

^2^As previously determined by van der Veer et al. (25).

iAMP21, intrachromosomal amplification of chromosome 21; No, number; IQR, interquartile range; WBC, white blood cell; HR, high risk; MRD, minimal residual disease.

### Common region of amplification

**Figure 1:**
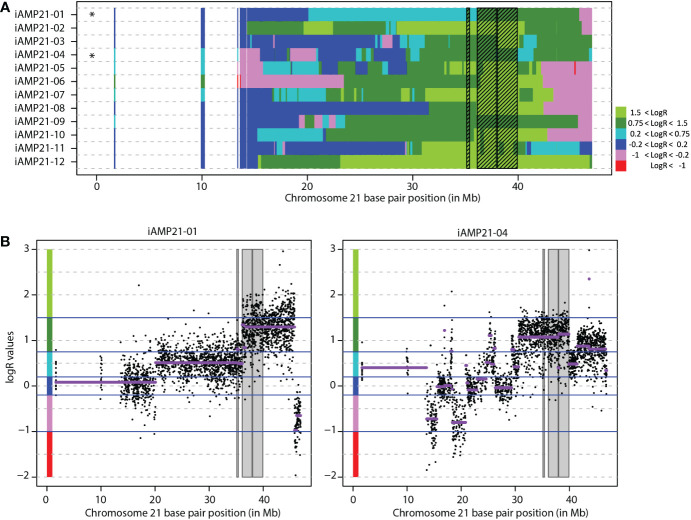
Determination of the common region of amplification in 12 iAMP21 cases. **(A)** Segmented chromosome 21 LogR values 12 copy number profiled iAMP21 cases. Each row depicts one case ordered randomly. Shaded region shows the common region of amplification in this cohort (CRA). Chromosome 21 base pair position is shown on the x-axis (reference genome hg18). Black stars depict cohort CRA defining cases. **(B)** Chromosome 21 copy number profile of the two cases that define the borders of the CRA. The y-axis shows normalized LogR values of individual probes (dots) and segmented LogR values (purple lines). Chromosome 21 base pair position is shown on the x-axis (reference genome hg18), with LogR value on the y-axis. Shaded region shows the CRA. Blue lines show the LogR value cut-offs as used in figure 1A, colours on the y-axis represent the colours as shown in figure 1A.

**Figure 2:**
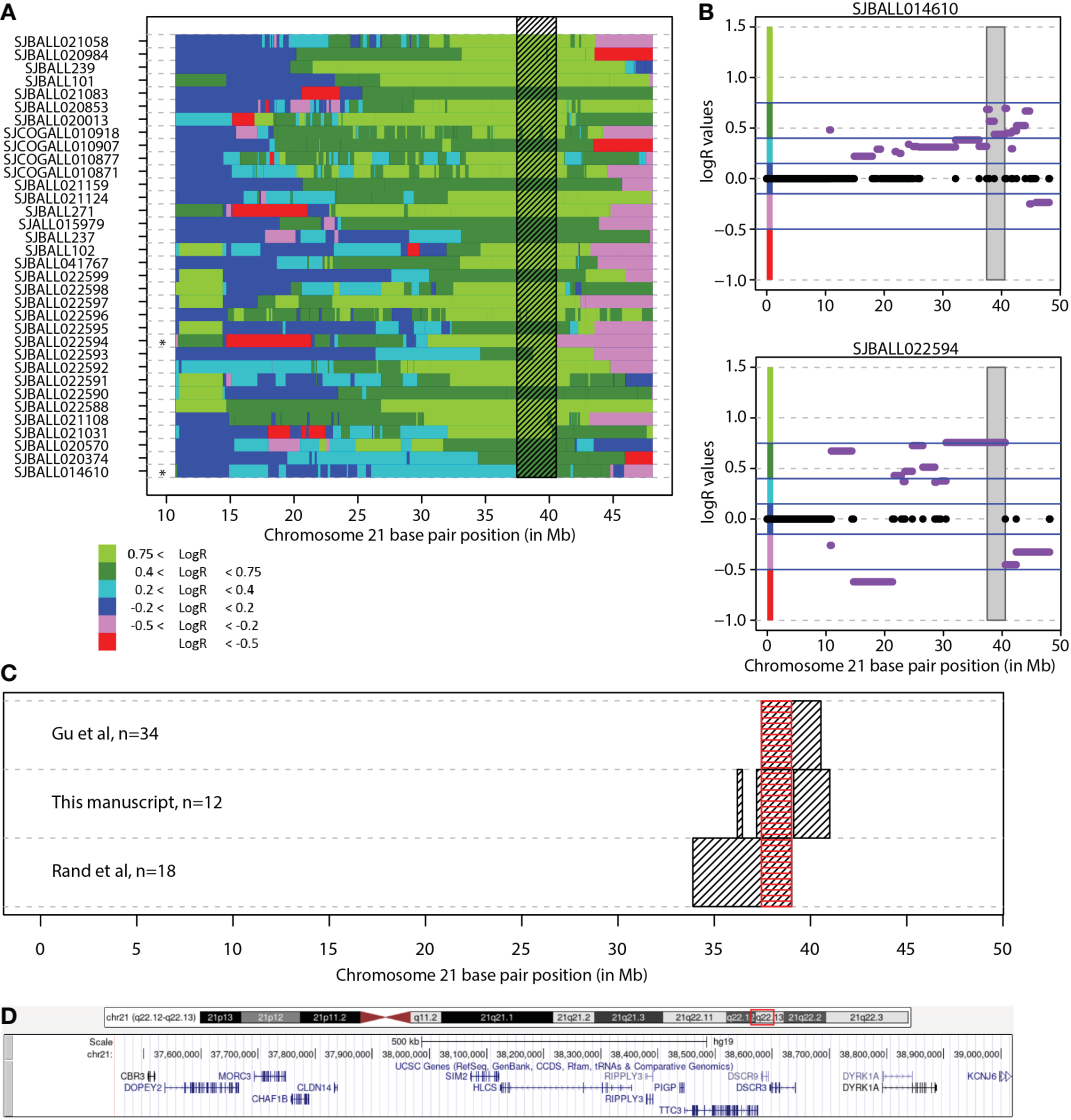
Combined common region of amplification. **(A)** Segmented chromosome 21 LogR values in 34 iAMP21 cases from the study of Gu et al. Each row depicts one case. Shaded region shows the common region of amplification in this cohort (CRA). Black stars depict cohort CRA defining cases. Chromosome 21 base pair position is shown on the x-axis (reference genome hg19). **(B)** Chromosome 21 copy number profile of the two cases from Gu et al. that define the region. Purple lines show the segMean values, values between -0.2-0.2 were absent from supplement, indicated in black. Chromosome 21 base pair position is shown on the x-axis (reference genome hg19), with LogR values on the y-axis. Shaded region shows the common region of amplification. Blue lines show the LogR value borders as used in figure 2A, colours on the y-axis represent the colours as shown in figure 2A. **(C)** The overlap of the CRA in all three studies. Black boxes show the region in each study, red box shows the overlap between all studies. Chromosome 21 base pair position is shown on the x-axis (reference genome hg19). **(D)** Overview common region of amplification. Snapshot taken from UCSC genome browser (Reference genome hg19/GRCh37; location chr21:37,452,074-39,019,555). *DSCR3* is also known as *VPS26C*.

The chromosome 21 copy number profile of the 12 iAMP21 cases showed amplifications mainly between 30 Mb to 40 Mb (**Supplementary Figure S2**). To determine the common region of amplification, a LogR cut-off of 0.75 was chosen based on comparison of aCGH profiles of high hyperdiploid samples (data not shown) and expected to indicate a copy number of four or greater. There were three regions where all 12 iAMP21 cases showed amplification, located from 35.11-35.38 Mb (containing *RUNX1*), 36.12-37.93 Mb, and 38.04-39.92 Mb (hg18; **Figures 1A, B**). To further define the CRA we extended our dataset to include the supplementary SNP array data of Gu et al. (24), where segMean values above 0.4 were determined to equal an amplification. Using the same strategy, the CRA in this cohort was defined, locating from 37.45-40.54 Mb (hg19; **Figures 2A, B**). By combining these two CRAs with the CRA as published by Rand (4), we defined a combined CRA based on 64 iAMP21 cases (**Figure 2C**). The combined CRA was located from 37.45-39.01 Mb (hg19; 36.07-37.64 Mb in the most recent GRCh38 genome build) and comprised of 12 protein-coding genes (*CBR3*, *DOPEY2*, *MORC3*, *CHAF1B*, *CLDN14*, *SIM2*, *HLCS*, *RIPPLY3* (also known as *DSCR6*), *PIGP*, *TTC3*, *DSCR3* (also known as *VPS26C*), and *DYRK1A*) and one non-coding gene (*DSCR9*; **Figure 2D**). Notably, both borders of the combined CRA seemed to be defined by two studies; our study and Rand et al. (4) at the centromeric border, our study and Gu et al. (24) at the telomeric border.

### Differential gene expression iAMP21 samples

To determine iAMP21-related genome-wide gene expression changes, we compared gene expression between 12 iAMP21 and 143 B-other samples (n = 143), excluding sentinel subtypes such as *ETV6-RUNX1* and high hyperdiploidy. As the B-other group is still a heterogeneous subgroup, the results should identify a unique iAMP21 effect. In total, 1126 probesets were differentially expressed, measuring 744 unique genes (**Supplementary Table S3**). Of these, 101 probesets, measuring 41 unique genes, were located on chromosome 21, while 1025 probesets, measuring 704 unique genes were located on other chromosomes. Most probesets were overexpressed, only five probesets on chromosome 21 were downregulated, while almost a third of probesets outside of chromosome 21 (307/1025 = 30%) were downregulated.

### *RUNX1* is an unlikely target of iAMP21

**Figure 3:**
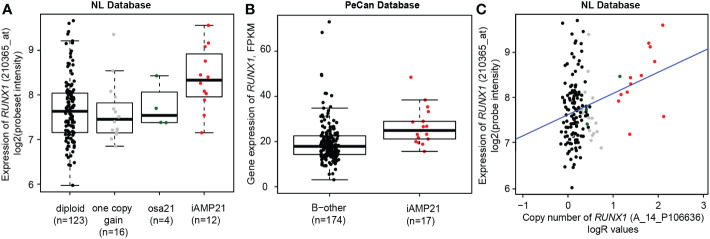
*RUNX1* copy number and expression in iAMP21 cases. **(A)** Expression levels of *RUNX1* (log2 intensities) categorized based on chromosome 21 copy number aberrations determined by aCGH: diploid, one copy gain, other structural aberration on chromosome 21 (osa21), and iAMP21. **(B)** Expression of *RUNX1* (FPKM) in the PeCan database. Selected cases were categorized based on iAMP21 status, as described in the PeCan database. **(C)** Correlation between copy number (normalized LogR values, x-axis) and gene expression (log2 values, y-axis). Cases are coloured based on chromosome 21 copy number aberrations determined by aCGH: diploid, one copy gain, other structural aberration on chromosome 21 (osa21), and iAMP21 (see figure 3A). Blue line represents the linear regression trendline using all samples shown in this figure.

*RUNX1*, located on chromosome 21, has often been mentioned as a potential oncogene affected by the transformation resulting in iAMP21. However, *RUNX1* is not located in the newly identified CRA. While expression of *RUNX1* was indeed modestly increased in iAMP21 compared with B-other in our dataset (log2(fold change) [logFC] = 0.69, FDR = 0.030; **Figure 3A**) and in the independent PeCan dataset (logFC = 0.44, Bonferroni corrected p-value = 0.002; **Figure 3B**), there was no correlation between *RUNX1* copy number and *RUNX1* gene expression (Bonferroni corrected p-value = 0.262, spearman correlation; **Figure 3C**).

### Prioritization of CRA genes

**Figure 4:**
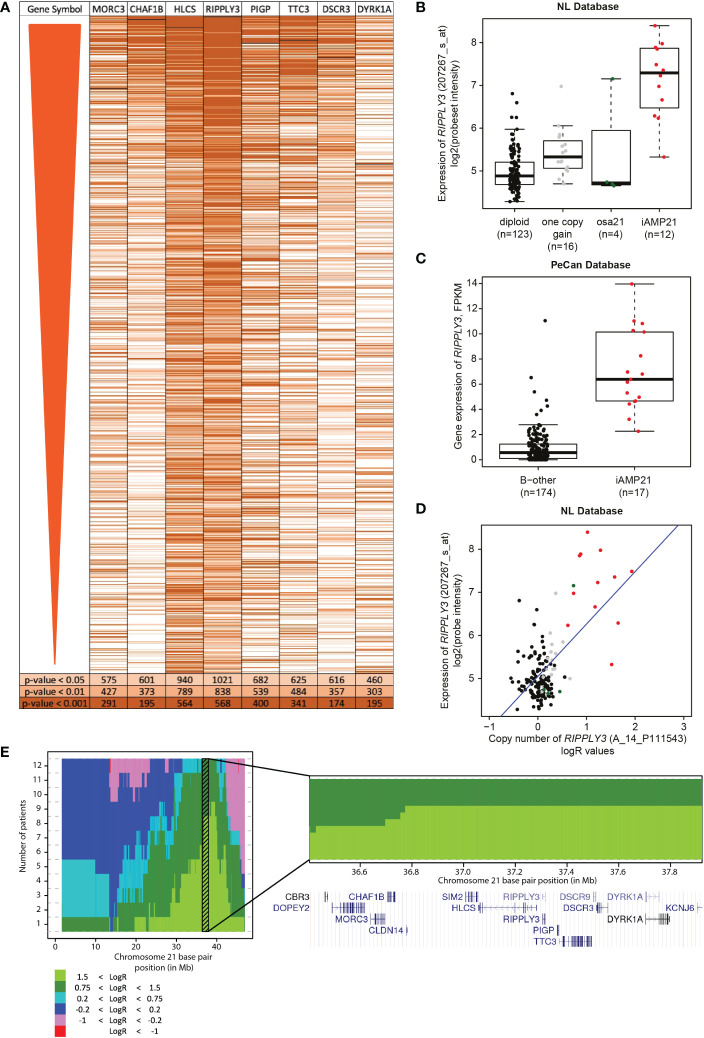
Correlation of *RIPPLY3* (also known as *DSCR6*) and the iAMP21 signature. **(A)** Heatmap showing the significant probesets in the differential gene expression analysis (FDR < 0.01; limma), ordered on FDR from lowest to high. Each row represents a probeset, colours show correlation of the probeset to one of the eight selected probesets, determined with gene-set testing (Globaltest). Light orange: local globaltest FDR < 0.05; medium orange: FDR < 0.01; dark orange: FDR < 0.001. **(B)** Expression levels of *RIPPLY3* (log2 intensities) categorized based on chromosome 21 copy number aberrations determined by aCGH: diploid, one copy gain, other structural aberration on chromosome 21 (osa21), and iAMP21. **(C)** Expression of *RIPPLY3* (FPKM) in the PeCan database. Selected cases are categorized based on iAMP21 status, as described in the PeCan database. **(D)** Correlation between copy number (normalized LogR values, x-axis) and gene expression (log2 values, y-axis). Cases are coloured based on chromosome 21 copy number aberrations determined by aCGH: diploid, one copy gain, other structural aberration on chromosome 21 (osa21), and iAMP21. Blue line represents the linear regression trendline using all samples shown in this figure. **(E)** Histogram showing the cumulative copy number in all 12 iAMP21 cases. Chromosome 21 base pair position is shown on the x-axis (reference genome hg18), number of cases on the y-axis. Colours show the segmented LogR values, see legend. Shaded region shows the combined CRA. Bottom panel shows a zoom-in of the combined CRA. Gene locations are indicated by the UCSC genome browser cut-out. *DSCR3* is also known as *VPS26C*.

iAMP21 leads to an increased copy number on chromosome 21, but the levels differ between cases. The genes affected by the transformation are likely located in the CRA, where the copy number gain is expected to result in increased gene expression. We prioritized the genes within the combined CRA based on three assumptions. Firstly, the gene should be overexpressed in iAMP21 samples compared with B-other samples. Of the 13 protein-coding genes in the combined CRA, eight (*MORC3*, *CHAF1B*, *HLCS*, *RIPPLY3*, *PIGP*, *TTC3*, *VPS26C*, and *DYRK1A*) were significantly overexpressed in iAMP21 cases compared with the non-iAMP21 B-other cases in our cohort and in the PeCan cohort (**Supplementary Table S4**; **Supplementary Figure S3**). Most of the eight selected genes were uniquely expressed in iAMP21 or increased in both iAMP21 and high hyperdiploid (not statistically tested, **Supplementary Figure S4**). Secondly, the copy number and gene expression should be correlated (cis-effect). The eight overexpressed genes in the CRA showed a positive correlation between copy number and gene expression (**Supplementary Table S4**; **Supplementary Figure S3**). These genes were either uniquely overexpressed in iAMP21 or increased in both iAMP21 and high hyperdiploid (not statistically tested, **Supplementary Figure S4**). Thirdly, we expect that genome-wide expression associated with the individual CRA gene expression as determined by gene-set testing with a non-supervised method (Globaltest) strongly overlaps with the iAMP21 signature (trans-effect). Each of the eight selected genes in the CRA was correlated to genome-wide gene expression changes (**Table 2**) and displayed highly different genome-wide effects, both in which genes were affected and in number of associated genes (**Supplementary Table S5**). The expression signature of *RIPPLY3* (also known as *DSCR6*), overlapped most with the iAMP21 differential gene expression results (**Figure 4A**). Of the 1126 iAMP21-related differentially expressed probesets, 838 (74%) were significantly associated with *RIPPLY3* expression, of which 759 were not located on chromosome 21. In addition, the -log10(p-values) of the iAMP21 signature showed the highest correlation with the *RIPPLY3* correlation signature (R = 0.58, **Table 2**). *RIPPLY3* was the highest overexpressed gene in the CRA of iAMP21 cases in our dataset (logFC = 2.08, FDR < 0.001; **Figure 4B**), as well as the PeCan dataset (logFC = 2.92, Bonferroni corrected p-value < 0.001; **Figure 4C**). In addition, *RIPPLY3* copy number and expression were highly correlated, with the steepest correlation of all genes tested (**Figure 4D**; **Table 2**). Interestingly, one sample with a total of 5 copies of the long arm of chromosome (osa21), showed high *RIPPLY3* expression as well (**Figure 4B**). *RIPPLY3* is located in the region with the highest amplification within the CRA (**Figure 4E**).

**Table 2:** Expression and genome-wide correlations of the eight selected genes in the combined iAMP21 common region of amplification.

		Differential gene expression NL^2^		Differential gene expression PeCan^3^		Correlation copy number *vs* expression^4^			Genome-wide signature^6^		
order in CRA^1^	gene symbol	logFC	FDR	logFC	Bonferroni p-value	Correlation coefficient (R)	Bonferroni p-value	Slope^5^	Number of significantly associated probesets^7^	Overlap with iAMP21 signature genes^8^	Correlation with iAMP21 signature^9^
3	*MORC3*	1.16	<0.001	1.13	<0.001	0.36	<0.001	0.68	18949	427/1126	-0.034
4	*CHAF1B*	1.03	<0.001	1.65	<0.001	0.36	<0.001	0.62	1186	373/1126	0.316
7	*HLCS*	0.61	<0.001	2.07	<0.001	0.37	<0.001	0.36	9737	789/1126	0.452
8	*RIPPLY3*	2.08	<0.001	2.92	<0.001	0.27	0.011	1.22	2331	838/1126	0.584
9	*PIGP*	1.59	<0.001	1.14	<0.001	0.33	<0.001	0.81	7918	539/1126	0.128
10	*TTC3*	1.58	<0.001	1.71	<0.001	0.46	<0.001	0.92	15581	484/1126	-0.009
12	*VPS26C*	0.50	<0.001	1.37	<0.001	0.32	<0.001	0.34	1396	357/1126	0.298
13	*DYRK1A*	1.04	<0.001	1.59	<0.001	0.37	<0.001	0.77	12655	303/1126	0.004

^1^Order in which genes are located in the combined CRA, from centromere to telomere. Numbers indicate the order considering all 13 genes.

^2^Comparing 12 iAMP21 versus 143 B-other samples using limma. All expression probesets on the Affymetrix U133 Plus 2 array were tested and false discovery rate was used for multiple testing correction.

^3^Comparing 17 iAMP21 samples with 174 B-other samples. FPKM expression values were extracted from the PeCan database. Differential expression was tested for the CRA genes using Mann Whitney U test and Bonferroni corrected.

^4^Spearman correlation of copy number with gene expression within each gene, using all 155 NL samples (B-other plus iAMP21).

^5^Slope of linear regression trendline using all 155 NL samples.

^6^Gene-set testing by Globaltest, which tests the association of gene expression of the indicated gene with genome-wide gene expression using 155 NL samples (not including iAMP21 status).

^7^Number of probesets significantly correlated with the expression of the CRA gene.

^8^Overlap between non-supervised genome-wide expression changes for the CRA gene and the iAMP21 limma signature (1126 probesets), showing number of probesets that significantly overlap with the iAMP21 signature.

^9^Spearman correlation coefficient (R) of -log10(FDR) in limma with local -log10(FDR) in Globaltest of all probesets.

CRA, common region of amplification; logFC, log 2 fold change; FDR, false discovery rate.

## Discussion

We narrowed down the CRA to 1.57 Mb, encompassing 13 genes, based on 64 cases. Of note, both borders of this CRA seemed to be defined by two studies and therefore by two different patients. While all CRA genes might be potential target genes of iAMP21, and could have a combined effect, *RIPPLY3* was highest prioritised based on genomic data integration. Since iAMP21 is a primary event in the development of BCP-ALL (4), it might confer its initiating effects *via RIPPLY3* and/or one of the other CRA genes.

A recent study from Öfverholm et al. (17) identified *MORC3*, *SON*, *CHAF1B* and *DYRK1A* as potential candidates for the iAMP21 pathophysiology. In our study, *MORC3*, *CHAF1B*, and *DYRK1A*, located in the CRA, were upregulated, but less strongly than *RIPPLY3* (*CHAF1B:* R = 0.32) or not at all correlated to the iAMP21 signature (*MORC3*: R = -0.034; *DYRK1A:* R = 0.004). *RIPPLY3* was the most overexpressed gene on chromosome 21 in their study, but not further evaluated since it was not significantly overexpressed in iAMP21 cases compared with high hyperdiploid cases. The study from Öfverholm et al. used a biased subset of cases, with 30% (14/46) dic (9, 20) cases, while we compared iAMP21 cases with population-based, diverse B-other cases. Although direct comparison is difficult, three out of four genes identified by this independent study were located in the combined CRA, indicating the potential specificity of this region.

*RIPPLY3* is a relatively understudied gene and no associations with leukaemia have been shown yet. Discovered in 2000 (33), RIPPLY3 plays an important role in embryonic development (34, 35). RIPPLY3 is a transcriptional corepressor, regulating the transcriptional activity of other transcription factors, such as TBX1 and STAT3 (36, 37). RIPPLY3, or the transcription factors it regulates, might affect gene expression changes observed in iAMP21 BCP-ALL, with 30% of the non-chromosome 21 differentially expressed genes being downregulated. We focussed on finding the target of the first step of the iAMP21 leukemic transformation, while other iAMP21 studies focus on the concomitant lesions, such as lesions in the RAS pathway (11) or the JAK/STAT pathway (12, 38). These lesions provide interesting targeting options using for example trametinib or ruxolitinib, however, they will only benefit cases with those specific lesions. Finding the gene or genes responsible for the BCP-ALL initiation, like *RIPPLY3* may be, might lead to the development of a more specific treatment, benefitting more or even all iAMP21 cases.

The role of chromosome 21 in paediatric BCP-ALL has been extensively studied in DS-ALL, since the risk of developing BCP-ALL is 20 times higher in children with a constitutional gain of chromosome 21 (39). DS is not always characterized by a gain of a complete chromosome 21 but is sometimes due to a partial trisomy of chromosome 21. Modelling the DS phenotype in mice has led to identification of a Down Syndrome Critical Region (DSCR), spanning 5.3 Mb and containing 33 genes (40). This region starts at the same position as the combined CRA. As shown by Lane et al. (41), triplication of this region leads to indefinite self-renewal of B-cell colonies. Lane et al. (41) identified *HMGN1*, located in the DSCR, as a candidate gene for DS BCP-ALL. *HMGN1* overexpression results in an increase in global transcription (42). Although overexpressed in our samples (LogFC = 0.95, Bonferroni corrected p-value < 0.001) *HMGN1* is located distal and hence outside the CRA at around 39.3 Mb (GRCh38).

Since iAMP21 is a high risk subtype, benefitting from a more intense treatment, accurate diagnosis of this subtype is essential (7, 10). In agreement with previous reports (43–45), we suggest that not all iAMP21 cases follow the current WHO definition that is focussed on *RUNX1* copy number. Although the distinctive genomic profile of chromosome 21 could be beneficial in identifying iAMP21 cases as well (46, 47), our identified CRA could aid in the diagnosis of iAMP21 based on copy number analysis. While *RUNX1* is likely not the target gene of iAMP21, it is still often in a region with high amplification, making the subgroup of iAMP21 patients with low *RUNX1* amplification small. It would be informative to compare clinical outcome in this small group of iAMP21 patients, that would be missed with classical FISH screening for *RUNX1*, with the remaining iAMP21 patients in an international collaboration.

This study presents an *in silico* approach to determine the potential target gene of iAMP21. We chose a cut-off that should represent a copy number of four or higher. As the same cut-off was used in all cases within the same cohort, this should accurately represent the CRA. In addition, we only used samples with a high blast percentage (above 90%), which minimizes the effect of normal cells on the LogR. While mRNA expression generally represents protein expression well, mRNA may not correlate with protein expression in all cases. A further determination of protein expression of genes in the CRA and functional studies of their role in leukaemia would be greatly beneficial in determining the potential target of iAMP21.

In summary, we further defined the CRA to a region of 1.57 Mb, located from 36.07 to 37.64 Mb (GRCh38) and identified *RIPPLY3* as the most prominent gene affected in iAMP21-positive BCP-ALL. As this region is amplified in all (studied) iAMP21 cases, it might be involved in the development of leukaemia as a result of iAMP21. Furthermore, this region might be of benefit in accurate diagnosis of iAMP21 BCP-ALL.

No drugs specifically targeting the deregulated gene expression in iAMP21 have been developed yet. Further studies should focus on studying the link between *RIPPLY3* and iAMP21 by performing functional *in vitro* studies. Identifying downstream pathways influenced by RIPPLY3 could benefit the development of more targeted treatment options of the high-risk iAMP21 subtype.

## Data availability statement

The data presented in the study are deposited in the Gene Expression Omnibus repository, accession number GSE87070 (gene expression) and GSE184692 (copy number).

## Author contributions

Contribution: The project was conceived by JB and MB and conceptualized together with FH. Experimental and computational analyses were performed by FH, AB and AH. Clinical samples and data were provided by ES and GE. Data interpretation was performed by FH, JM, and MB. The manuscript was drafted by FH, JB and MB. All authors contributed to the article and approved the submitted version.
