# Supplementary material for: Integrating copy number data of 64 iAMP21 BCP-ALL patients narrows the common region of amplification to 1.57 Mb
Source: Front Oncol. 2023 Feb 23;13:1128560. doi: 10.3389/fonc.2023.1128560 (PMC9996016; doi:10.3389/fonc.2023.1128560)
Supplement: Supplementary file 1 [file DataSheet_1.pdf]

Supplementary information for:

ARTICLE TITLE

Integrating copy number data of 64 iAMP21 BCP-ALL patients narrows the common region of amplification to 1.57 Mb

AUTHORS

Femke M. Hormann[1,2,3], Alex Q. Hoogkamer[1,2], Aurélie Boeree[1,2], Edwin Sonneveld[1,4], Gabriele Escherich[5,6], Monique L. den Boer[1,2,3,4], Judith M. Boer[1,2].

[1] Princess Máxima Center for pediatric oncology, Utrecht, Netherlands.

[2] Oncode Institute, Utrecht, Netherlands.

[3] Erasmus MC - Sophia Children's Hospital, Department of Pediatric Oncology and Hematology, Rotterdam, Netherlands.

[4] Dutch Childhood Oncology Group, Utrecht, Netherlands

[5] COALL – German Cooperative Study Group for Childhood Acute Lymphoblastic Leukemia, Hamburg, Germany

[6] Clinic of Pediatric Hematology and Oncology, University Medical Center Hamburg-Eppendorf, Hamburg, Germany

CONTENTS

Tables:

- Supplementary Table S1: GEO identifiers of selected patients
- Supplementary Table S2: Patient characteristics of 12 iAMP21 patients
- Supplementary Table S3: Top 100 significantly differentially expressed probesets between 12 iAMP21 and 143 B-other cases, determined by Limma
- Supplementary Table S4: Differential gene expression of all 13 genes in the CRA and correlation gene expression with copy number
- Supplementary Table S5: Top 50 most correlated expression probesets to the eight selected genes in the CRA

Figures:

- Supplementary Figure S1: Selection of the samples
- Supplementary Figure S2: Chromosome 21 copy number profile in all 12 iAMP21 patients
- Supplementary Figure S3: Gene expression levels and correlation with copy number of all 13 CRA genes
- Supplementary Figure S4: Gene expression levels of the 8 selected CRA genes in all main subtypes

Supplementary Table S1: GEO identifiers of selected patients			
Samplename in manuscript	samplename in GEO	chromosome 21 group	additional subtype information
iAMP21-01	A38	iAMP21	BCR-ABL1-like
iAMP21-02	A114	iAMP21	BCR-ABL1-like
iAMP21-03	A170	iAMP21	BCR-ABL1-like
iAMP21-04	A372	iAMP21	BCR-ABL1-like
iAMP21-05	A397	iAMP21	BCR-ABL1-like
iAMP21-06	A403	iAMP21	BCR-ABL1-like
iAMP21-07	A411	iAMP21	BCR-ABL1-like
iAMP21-08	A458	iAMP21	BCR-ABL1-like
iAMP21-09	A470	iAMP21	BCR-ABL1-like
iAMP21-10	A200	iAMP21	B-other
iAMP21-11	A224	iAMP21	BCR-ABL1-like
iAMP21-12	A426	iAMP21	BCR-ABL1-like
remaining B-other	A42	diploid	BCR-ABL1-like
remaining B-other	R83	diploid	BCR-ABL1-like
remaining B-other	A135	diploid	BCR-ABL1-like
remaining B-other	A10	diploid	BCR-ABL1-like
remaining B-other	A101	diploid	BCR-ABL1-like
remaining B-other	A15	diploid	BCR-ABL1-like
remaining B-other	A34	diploid	BCR-ABL1-like
remaining B-other	A41	diploid	BCR-ABL1-like
remaining B-other	A77	diploid	BCR-ABL1-like
remaining B-other	A35	diploid	BCR-ABL1-like
remaining B-other	A251	diploid	BCR-ABL1-like
remaining B-other	A428	diploid	BCR-ABL1-like
remaining B-other	A472	diploid	BCR-ABL1-like
remaining B-other	R32	diploid	BCR-ABL1-like
remaining B-other	A214	diploid	BCR-ABL1-like
remaining B-other	A216	diploid	BCR-ABL1-like
remaining B-other	A146	diploid	BCR-ABL1-like
remaining B-other	A188	diploid	BCR-ABL1-like
remaining B-other	A208	diploid	BCR-ABL1-like
remaining B-other	A225	diploid	BCR-ABL1-like
remaining B-other	A253	diploid	BCR-ABL1-like
remaining B-other	A30	diploid	BCR-ABL1-like
remaining B-other	A314	diploid	BCR-ABL1-like
remaining B-other	A32	diploid	BCR-ABL1-like
remaining B-other	A40	diploid	BCR-ABL1-like
remaining B-other	A407	diploid	BCR-ABL1-like
remaining B-other	A513	diploid	BCR-ABL1-like
remaining B-other	A529	diploid	BCR-ABL1-like
remaining B-other	A82	diploid	BCR-ABL1-like
remaining B-other	A92	diploid	BCR-ABL1-like
remaining B-other	R44	diploid	BCR-ABL1-like
remaining B-other	A204	diploid	BCR-ABL1-like
remaining B-other	A31	diploid	BCR-ABL1-like
remaining B-other	A20	diploid	BCR-ABL1-like
remaining B-other	A108	diploid	BCR-ABL1-like
remaining B-other	A148	diploid	BCR-ABL1-like
remaining B-other	A150	diploid	BCR-ABL1-like
remaining B-other	A190	diploid	BCR-ABL1-like
remaining B-other	A249	diploid	BCR-ABL1-like
remaining B-other	A505	diploid	BCR-ABL1-like
remaining B-other	A74	diploid	BCR-ABL1-like
remaining B-other	A87	diploid	BCR-ABL1-like
remaining B-other	A117	diploid	BCR-ABL1-like
remaining B-other	A526	diploid	BCR-ABL1-like
remaining B-other	A393	diploid	BCR-ABL1-like
remaining B-other	A91	diploid	BCR-ABL1-like
remaining B-other	A26	diploid	BCR-ABL1-like

remaining B-other	A430	diploid	BCR-ABL1-like
remaining B-other	A48	diploid	BCR-ABL1-like
remaining B-other	A89	diploid	BCR-ABL1-like
remaining B-other	A96	diploid	BCR-ABL1-like
remaining B-other	R35	diploid	BCR-ABL1-like
remaining B-other	R91	diploid	BCR-ABL1-like
remaining B-other	A420	diploid	BCR-ABL1-like
remaining B-other	A178	diploid	B-other
remaining B-other	A104	diploid	B-other
remaining B-other	A425	diploid	B-other
remaining B-other	A93	diploid	B-other
remaining B-other	A167	diploid	B-other
remaining B-other	A194	diploid	B-other
remaining B-other	A199	diploid	B-other
remaining B-other	A232	diploid	B-other
remaining B-other	A97	diploid	B-other
remaining B-other	A221	diploid	B-other
remaining B-other	A99	diploid	B-other
remaining B-other	A1	diploid	B-other
remaining B-other	A103	diploid	B-other
remaining B-other	A11	diploid	B-other
remaining B-other	A110	diploid	B-other
remaining B-other	A116	diploid	B-other
remaining B-other	A124	diploid	B-other
remaining B-other	A126	diploid	B-other
remaining B-other	A136	diploid	B-other
remaining B-other	A154	diploid	B-other
remaining B-other	A166	diploid	B-other
remaining B-other	A174	diploid	B-other
remaining B-other	A197	diploid	B-other
remaining B-other	A198	diploid	B-other
remaining B-other	A22	diploid	B-other
remaining B-other	A347	diploid	B-other
remaining B-other	A417	diploid	B-other
remaining B-other	A421	diploid	B-other
remaining B-other	A440	diploid	B-other
remaining B-other	A47	diploid	B-other
remaining B-other	A485	diploid	B-other
remaining B-other	A49	diploid	B-other
remaining B-other	A494	diploid	B-other
remaining B-other	A497	diploid	B-other
remaining B-other	A521	diploid	B-other
remaining B-other	A535	diploid	B-other
remaining B-other	A537	diploid	B-other
remaining B-other	A538	diploid	B-other
remaining B-other	A541	diploid	B-other
remaining B-other	A68	diploid	B-other
remaining B-other	A71	diploid	B-other
remaining B-other	A94	diploid	B-other
remaining B-other	R10	diploid	B-other
remaining B-other	R48	diploid	B-other
remaining B-other	R59	diploid	B-other
remaining B-other	R60	diploid	B-other
remaining B-other	R9	diploid	B-other
remaining B-other	A111	diploid	B-other
remaining B-other	A121	diploid	B-other
remaining B-other	A469	diploid	B-other
remaining B-other	A2	diploid	B-other
remaining B-other	A348	diploid	B-other
remaining B-other	A69	diploid	B-other
remaining B-other	A95	diploid	B-other
remaining B-other	A144	diploid	B-other
remaining B-other	A165	diploid	B-other

remaining B-other	A483	diploid	B-other
remaining B-other	A54	diploid	B-other
remaining B-other	A76	diploid	B-other
remaining B-other	A85	diploid	B-other
remaining B-other	A367	diploid	B-other
remaining B-other	A100	diploid	B-other
remaining B-other	A254	diploid	B-other
remaining B-other	A422	diploid	B-other
remaining B-other	A102	diploid	B-other
remaining B-other	A448	diploid	B-other
remaining B-other	A83	diploid	B-other
remaining B-other	A84	diploid	B-other
remaining B-other	A72	diploid	B-other
remaining B-other	A70	one copy gain	BCR-ABL1-like
remaining B-other	A123	one copy gain	BCR-ABL1-like
remaining B-other	A160	one copy gain	B-other
remaining B-other	A234	one copy gain	B-other
remaining B-other	A171	one copy gain	B-other
remaining B-other	A223	one copy gain	B-other
remaining B-other	A235	one copy gain	B-other
remaining B-other	A39	one copy gain	B-other
remaining B-other	A459	one copy gain	B-other
remaining B-other	A88	one copy gain	B-other
remaining B-other	A19	one copy gain (DS)	BCR-ABL1-like
remaining B-other	A424	one copy gain (DS)	BCR-ABL1-like
remaining B-other	A287	one copy gain (DS)	B-other
remaining B-other	A14	one copy gain (DS)	B-other
remaining B-other	A359	one copy gain (DS)	B-other
remaining B-other	A156	one copy gain (DS)	B-other
remaining B-other	A81	osa21	BCR-ABL1-like
remaining B-other	A282	osa21	B-other
remaining B-other	A141	osa21	B-other
remaining B-other	A481	osa21	B-other

Abbreviations: iAMP21, intrachromosomal amplification of chromosome 21; DS, Down syndrome; osa21, other structural aberration of chromosome 21

Supplementary Table S2: Patient characteristics of 12 iAMP21 patients									
	gender	age (years)	WBC ($x10^9/L$)	Protocol-risk arm	MRD TP1	MRD TP2	Outcome (years)	Down syndrome	karyotype
iAMP21-01	M	6	128.8	ALL9-HR	-	-	CCR 8.35	No	46, XY[4]
iAMP21-02	F	7	11	COALL97-HR	-	-	CCR 7.47	No	-
iAMP21-03	F	12	14.5	COALL03-HR	0.000001	-	CCR 4.64	No	-
iAMP21-04	F	7	2	ALL10-MR	0.0019	0.00027	relapse 2.88	No	-
iAMP21-05	M	12	1.5	ALL10-MR	0.002	0	relapse 3.91	No	46, -X, -Y, -2, -5, del(6)(q21q23), -12, -13, -17, -18, -21, i(21)(q10), +10mar[cp13] / 46, XY[4]
iAMP21-06	M	5	4.5	ALL10-MR	0.0001	0.00004	relapse 3.17	Yes	46, XY, ?t(13;20)(q1?4;q1?3), der(21)r(21).ishder(21)amp(21)(q22)r(21)(WCP21+,AML1++++)[4] / 46, XY, der(21)dup(21)(p11.1q22.3)r(21) (p11.1q22.3)c[6]
iAMP21-07	M	9	8	ALL10-SR	0	0	relapse 2.73	No	46, XY, -21, +mar, inc[7] / 46, XY[13]
iAMP21-08	M	5	21	ALL10-HR	0.004	0.000001	relapse 2.42	No	46, XY, ?8q, add(21)(q22), inc[8] / 46, XY[2]
iAMP21-09	F	8	7.2	ALL10-MR	0.000001	0	CCR 4.83	No	46, XX, dup(21)(q22)[10] / 46, XX[6]
iAMP21-10	M	10	50	COALL03-HR	0	-	CCR 3.69	No	46, XY, -21, +r, inc / 46, XY, idem, -?8, +mar
iAMP21-11	M	12	3.8	COALL97-HR	0.27	-	CCR 8.84	No	-
iAMP21-12	F	14	2.9	ALL10-MR	0.00017	0	CCR 5.77	No	46, XX, del(10)(q22q26), del(13)(q1?3q34), dup(21)(q22)[4] / 46, XX[18]

Abbreviations: M, male; F, female; WBC, white blood cell count; MRD, minimal residual disease; TP, time point; CCR, continuous complete remission; HR, high risk; MR, medium risk; SR, standard risk

Supplementary Table S3: Top 100 significantly differentially expressed probesets between 12 iAMP21 and 143 B-other cases, determined by Limma.

Probeset	Gene Symbol	Entrez gene ID	Chromosome	logFC[1]	FDR[2]
38487_at	STAB1	23166	3	3.30	4.80E-24
204150_at	STAB1	23166	3	3.24	2.75E-22
**207267_s_at**	**RIPPLY3**	**53820**	**21**	**2.08**	**2.82E-21**
204979_s_at	SH3BGR	6450	21	0.89	3.35E-19
227832_at	MBD6	114785	12	0.74	3.35E-19
232165_at	EPPK1	83481	8	1.09	2.96E-17
**204775_at**	**CHAF1B**	**8208**	**21**	**1.03**	**3.74E-15**
223967_at	ANGPTL6	83854	19	0.93	3.74E-15
211410_x_at	KIR2DL5A	57292	19	1.07	4.85E-15
232164_s_at	EPPK1	83481	8	1.41	1.02E-14
213715_s_at	KANK3	256949	19	0.94	1.16E-14
232934_at	---	---	21	0.88	1.32E-14
228001_at	TMEM50B	757	21	0.98	4.19E-14
207313_x_at	KIR3DL2 /// LOC727787	3812 /// 727787	19	1.53	6.49E-14
216907_x_at	KIR3DL2 /// LOC727787	3812 /// 727787	19	1.93	7.50E-14
205987_at	CD1C	911	1	2.29	1.46E-13
211688_x_at	KIR3DL2 /// LOC727787	3812 /// 727787	19	1.97	1.71E-13
229698_at	---	---	22	0.94	2.14E-13
207314_x_at	KIR3DL2 /// LOC727787	3812 /// 727787	19	2.58	2.89E-13
211163_s_at	TNFRSF10C	8794	8	0.85	7.33E-13
**209399_at**	**HLCS**	**3141**	**21**	**0.61**	**8.41E-13**
205858_at	NGFR	4804	17	1.32	1.17E-12
218950_at	ARAP3	64411	5	0.61	2.69E-12
211687_x_at	KIR3DL1	3811	19	0.90	9.67E-12
222907_x_at	TMEM50B	757	21	1.04	1.24E-11
201086_x_at	SON	6651	21	1.83	1.64E-11
238071_at	LCN10	414332	9	2.11	2.17E-11
1554852_a_at	KIAA1257	57501	3	0.50	2.82E-11
216552_x_at	KIR2DS4	3809	19	0.62	3.19E-11
230861_at	DKFZP434L187	26082	15	1.21	3.53E-11
1553297_a_at	CSF3R	1441	1	1.96	4.07E-11
203591_s_at	CSF3R	1441	1	2.22	4.30E-11
203405_at	PSMG1	8624	21	1.54	6.22E-11
214988_s_at	SON	6651	21	1.87	9.93E-11
201494_at	PRCP	5547	11	-1.18	1.11E-10
211389_x_at	KIR3DS1	3813	19	0.66	1.39E-10
1557329_at	---	---	21	0.47	1.89E-10
206400_at	LGALS7 /// LGALS7B	3963 /// 653499	19	1.04	2.02E-10
1554767_s_at	CRYZL1	9946	21	1.12	3.01E-10
211597_s_at	HOPX	84525	4	2.48	3.05E-10
1552347_at	CRYZL1	9946	21	1.57	3.18E-10
213989_x_at	SETD4	54093	21	0.77	3.52E-10
**208662_s_at**	**TTC3**	**7267**	**21**	**1.58**	**4.17E-10**
**208661_s_at**	**TTC3**	**7267**	**21**	**1.69**	**4.62E-10**
200944_s_at	HMGN1	3150	15	0.95	5.39E-10
225182_at	TMEM50B	757	21	1.26	5.39E-10
223407_at	C16orf48	84080	16	1.07	5.83E-10
**221689_s_at**	**PIGP**	**51227**	**21**	**1.59**	**6.58E-10**
244622_at	BRWD1	54014	21	0.70	9.86E-10
208203_x_at	KIR2DS5	3810	19	0.68	1.71E-09
226465_s_at	SON	6651	21	1.23	1.73E-09
1559679_a_at	---	---	12	0.55	2.03E-09
205254_x_at	TCF7	6932	5	1.26	2.11E-09
239602_at	---	---	21	0.69	2.25E-09

244840_x_at	DOCK4	9732	7	0.96	2.76E-09
202855_s_at	SLC16A3	9123	17	0.79	3.07E-09
**208073_x_at**	**TTC3**	**7267**	**21**	**1.57**	**3.71E-09**
205255_x_at	TCF7	6932	5	1.23	4.48E-09
202749_at	WRB	7485	21	1.54	5.43E-09
208179_x_at	KIR2DL3	3804	19	0.69	5.89E-09
207550_at	MPL	4352	1	0.94	6.15E-09
228067_at	C2orf55	343990	2	1.61	7.63E-09
220006_at	CCDC48	79825	3	0.48	7.93E-09
226071_at	ADAMTSL4	54507	1	0.84	9.87E-09
202856_s_at	SLC16A3	9123	12	1.39	1.03E-08
202993_at	ILVBL	10994	19	0.75	1.06E-08
**210645_s_at**	**TTC3**	**7267**	**21**	**1.57**	**1.06E-08**
200943_at	HMGN1	3150	15	0.92	1.09E-08
213779_at	EMID1	129080	22	0.97	1.31E-08
204468_s_at	TIE1	7075	1	1.39	1.37E-08
227424_x_at	C21orf119	84996	21	0.76	1.37E-08
225203_at	PPP1R16A	84988	8	0.96	1.39E-08
**217309_s_at**	**VPS26C**	**10311**	**21**	**0.50**	**1.54E-08**
1569476_at	DKFZP434L187	26082	15	0.55	1.80E-08
219600_s_at	TMEM50B	757	21	1.14	1.81E-08
200818_at	ATP5O	539	21	0.93	1.90E-08
225628_s_at	MLLT6	4302	17	0.74	1.93E-08
243091_at	---	---	13	0.72	2.15E-08
219482_at	SETD4	54093	21	0.95	2.21E-08
227833_s_at	MBD6	114785	12	1.23	2.21E-08
240063_at	LOC441046	441046	11	0.48	2.21E-08
216676_x_at	KIR3DL3	115653	19	0.79	2.28E-08
230097_at	GART	2618	21	1.28	3.01E-08
242579_at	BMPR1B	658	4	1.58	3.70E-08
223693_s_at	RADIL	55698	7	0.73	3.92E-08
214820_at	BRWD1	54014	21	1.04	4.09E-08
212996_s_at	URB1	9875	21	0.79	4.10E-08
1560981_a_at	PPARA	5465	22	0.82	4.32E-08
211250_s_at	SH3BP2	6452	4	0.75	4.61E-08
210128_s_at	LTB4R	1241	14	0.75	5.89E-08
211532_x_at	KIR2DS1 /// KIR2DS2 /// KIR2DS4	100132285 /// 3806 /// 3809	19	0.88	6.32E-08
229967_at	CMTM2	146225	16	1.97	6.76E-08
214745_at	PLCH1	23007	3	1.41	7.55E-08
221529_s_at	PLVAP	83483	19	1.64	8.18E-08
233753_at	SFRS15	57466	21	1.10	8.18E-08
236969_at	---	---	21	0.63	8.77E-08
**1569472_s_at**	**TTC3**	**7267**	**21**	**1.31**	**9.15E-08**
212358_at	CLIP3	25999	19	0.70	1.29E-07
216388_s_at	LTB4R	1241	14	0.62	1.47E-07
219722_s_at	GDPD3	79153	16	0.48	1.47E-07

[1] Log2 fold change iAMP21 cases (n=12) versus B-other ALL cases (n=143), genes in green are overexpressed, genes in red are underexpressed in iAMP21. Genes in bold are located in the CRA

[2] Genes are ordered on FDR, ascending, only top 100 are shown

Abbreviations: LogFC, Log 2 fold change; CRA, common region of amplification; FDR, false discovery rate.

Supplementary Table S4: Differential gene expression of all 13 genes in the CRA and correlation gene expression with copy number

General						Differential gene expression NL[3]			Differential gene expression PeCan[4]		Correlation copy number vs expression[5]		
Order[1]	gene	Entrez gene ID	Probeset expression array	Copy number probe	CN probe start location[2]	Mean exp. (log2 int.)	logFC	FDR	logFC	Bonf. p-value	Corr. Coefficient (R)	Bonf. p-value	Slope[6]
1	*CBR3*	874	205379_at	A_14_P104798	36440202	7.0	0.28	0.274	0.80	0.379	0.08	1.000	0.14
2	*DOPEY2*	9980	205248_at	A_16_P03583074	36466945	6.0	0.38	0.051	1.06	<0.001	0.22	0.082	0.35
3	*MORC3*	23515	213000_at	A_16_P41444361	36669380	8.0	1.16	<0.001	1.13	<0.001	0.36	<0.001	0.68
4	*CHAF1B*	8208	204775_at	A_16_P21247086	36696782	7.1	1.03	<0.001	1.65	<0.001	0.36	<0.001	0.62
5	*CLDN14*	23562	210689_at	A_16_P21247408	36823745	5.4	0.01	0.972	1.32	0.040	-0.16	0.601	-0.03
6	*SIM2*	6493	208157_at	A_16_P21247903	37011325	5.8	0.13	0.042	2.04	<0.001	0.28	0.006	0.09
7	*HLCS*	3141	209399_at	A_14_P106854	37047246	6.3	0.61	<0.001	2.07	<0.001	0.37	<0.001	0.36
8	*RIPPLY3*	53820	207267_s_at	A_14_P111543	37307755	5.2	2.08	<0.001	2.92	<0.001	0.27	0.012	1.22
9	*PIGP*	51227	221689_s_at	A_16_P03584371	37364124	9.1	1.59	<0.001	1.14	<0.001	0.33	<0.001	0.81
10	*TTC3*	7267	1569472_s_at	A_16_P21249052	37477504	10.1	1.58	<0.001	1.71	<0.001	0.46	<0.001	0.92
11	*DSCR9*	257203	230470_at	A_16_P03584581	37511631	6.4	0.16	0.032	NA	NA	0.05	1.000	0.11
12	*VPS26C*	10311	203635_at	A_16_P21249225	37538691	6.0	0.50	<0.001	1.37	<0.001	0.32	0.001	0.34
13	*DYRK1A*	1859	209033_s_at	A_16_P41447023	37702942	10.4	1.04	<0.001	1.59	<0.001	0.37	<0.001	0.77

[1] Order in which genes are located in the combined CRA, from centromere to telomere
[2] Start location of the copy number probe, reference genome is hg18
[3] Comparing 12 iAMP21 versus 143 non-iAMP21 B-other samples. All expression probesets on the Affymetrix U133 Plus 2 array are tested, and FDR is used for multiple testing correction.
[4] Comparing 17 iAMP21 samples with 174 B-other samples. FPKM expression values were extracted from the PeCan database. Differential expression is tested using Mann Whitney U test, multiple testing is applied to the 13 tests by Bonferroni
[5] Spearman correlation of copy number with gene expression within each gene, using 12 iAMP21 and 143 B-other samples (not including iAMP21 status in test).
[6] Slope of linear regression trendline using all 155 B-other samples.
Abbreviations: CRA, common region of amplification; CN, copy number; log2 int., log 2 intensities; logFC, log 2 fold change; FDR, false discovery rate; Bonf. P-value, Bonferroni corrected p-value; Corr. coefficient, correlation coefficient

Supplementary Table S5: Top 50 most correlated expression probesets to the eight selected genes in the CRA.

MORC3 = 213000_at			CHAF1B = 204775_at			HLCS = 209399_at			RIPPLY3 = 207267_s_at		
probeset	Gene symbol	FDR	probeset	Gene symbol	FDR	probeset	Gene symbol	FDR	probeset	Gene symbol	FDR
1553227_s_at	BRWD1	4.8E-24	221677_s_at	DONSON	3.9E-19	232934_at	---	1.9E-17	232934_at	---	7.5E-15
212990_at	SYNJ1	1.3E-23	203405_at	PSMG1	7.0E-14	217309_s_at	VPS26C	4.2E-17	1557329_at	---	1.2E-13
227428_at	GABPA	1.8E-23	219829_at	ITGB1BP2	8.3E-11	207833_s_at	HLCS	3.5E-16	205987_at	CD1C	1.2E-13
219280_at	BRWD1	1.8E-23	207267_s_at	RIPPLY3	9.8E-11	1557329_at	---	3.6E-14	38487_at	STAB1	1.3E-13
225153_at	GFM1	2.1E-23	201755_at	MCM5	1.0E-10	206564_at	OPRL1	5.1E-13	229967_at	CMTM2	2.2E-12
231960_at	BRWD1	5.8E-22	237005_at	LOC442075	1.2E-10	206222_at	TNFRSF10C	5.9E-12	214745_at	PLCH1	2.2E-12
215596_s_at	RNF160	5.8E-22	216237_s_at	MCM5	1.2E-10	217055_x_at	---	6.1E-12	222907_x_at	TMEM50B	2.2E-12
229317_at	KPNA5	7.1E-22	221521_s_at	GINS2	1.2E-10	225824_at	CCNK	8.3E-12	204304_s_at	PROM1	2.2E-12
227934_at	KPNA5	6.7E-21	200944_s_at	HMGN1	3.6E-10	211163_s_at	TNFRSF10C	1.9E-11	238488_at	LRRC70	3.9E-12
217886_at	EPS15	8.1E-21	200642_at	SOD1	5.6E-10	210332_at	LOC100134498	6.6E-11	227832_at	MBD6	1.5E-11
214820_at	BRWD1	1.8E-20	200943_at	HMGN1	1.3E-09	233840_at	LOC100130950	1.5E-10	204150_at	STAB1	2.4E-11
214753_at	N4BP2L2	1.8E-20	213135_at	TIAM1	1.3E-09	223718_at	ACRBP	1.6E-10	204775_at	CHAF1B	3.3E-11
238119_at	---	1.8E-20	200818_at	ATP5O	1.3E-09	205581_s_at	NOS3	2.1E-10	230718_at	HSF5	5.7E-11
214988_s_at	SON	4.2E-20	242890_at	---	1.3E-09	229191_at	TBCD	2.4E-10	232165_at	EPPK1	6.2E-11
235432_at	NPHP3	5.0E-20	205858_at	NGFR	2.2E-09	208980_s_at	UBC	3.2E-10	213989_x_at	SETD4	6.3E-11
232297_at	---	8.3E-20	204483_at	ENO3	2.3E-09	219075_at	YIPF2	3.3E-10	226071_at	ADAMTSL4	1.4E-10
226128_at	---	1.1E-19	202107_s_at	MCM2	4.1E-09	231710_at	CAPS	4.0E-10	203591_s_at	CSF3R	1.5E-10
239778_x_at	---	1.4E-19	1553297_a_at	CSF3R	5.2E-09	207267_s_at	RIPPLY3	4.6E-10	232164_s_at	EPPK1	2.9E-10
201086_x_at	SON	1.9E-19	205909_at	POLE2	5.4E-09	206072_at	UCN	4.9E-10	1553297_a_at	CSF3R	3.1E-10
221899_at	N4BP2L2	2.6E-19	223274_at	TCF19	5.4E-09	220130_x_at	LTB4R2	5.4E-10	219600_s_at	TMEM50B	3.3E-10
225158_at	GFM1	4.6E-19	217309_s_at	VPS26C	7.2E-09	239947_at	---	5.5E-10	209399_at	HLCS	3.9E-10

226337_at	GORAB	8.2E-19	1554696_s_at	TYMS	7.2E-09	38487_at	STAB1	5.8E-10	225182_at	TMEM50B	4.7E-10
212149_at	EFR3A	8.8E-19	1554767_s_at	CRYZL1	9.4E-09	238051_x_at	PWWP2B	6.3E-10	202993_at	ILVBL	5.1E-10
212795_at	KIAA1033	1.3E-18	222777_s_at	WHSC1	9.4E-09	232164_s_at	EPPK1	6.5E-10	1568795_at	---	5.7E-10
226508_at	PHC3	2.0E-18	203591_s_at	CSF3R	1.4E-08	229917_at	AGAP2	6.5E-10	1552665_at	LOC84989	5.8E-10
202797_at	SACM1L	2.1E-18	202589_at	TYMS	1.5E-08	205458_at	MC1R	6.5E-10	225681_at	CTHRC1	9.0E-10
213743_at	CCNT2	3.2E-18	209399_at	HLCS	1.8E-08	239386_at	---	6.5E-10	220059_at	STAP1	9.3E-10
228751_at	CLK4	3.2E-18	239253_at	---	3.0E-08	210099_at	ABCA2	6.5E-10	223967_at	ANGPTL6	1.7E-09
236196_at	---	3.3E-18	1564482_at	ATP5O	3.2E-08	232498_at	HEATR7A	7.3E-10	202855_s_at	SLC16A3	2.0E-09
226601_at	SLC30A7	4.2E-18	203976_s_at	CHAF1A	3.8E-08	234711_s_at	BCORL1	7.5E-10	228001_at	TMEM50B	2.5E-09
204194_at	BACH1	5.0E-18	205053_at	PRIM1	4.4E-08	1566501_at	---	7.9E-10	227833_s_at	MBD6	3.2E-09
234982_at	UBR3	5.7E-18	203975_s_at	CHAF1A	4.5E-08	241609_at	FOXD3	7.9E-10	229645_at	C18orf51	3.3E-09
225814_at	XRN1	5.7E-18	227832_at	MBD6	7.5E-08	230833_at	ACRBP	7.9E-10	209500_x_at	TNFSF12-TNFSF13 /// TNFSF13	7.6E-09
221905_at	CYLD	5.7E-18	232934_at	---	1.0E-07	236186_x_at	IL17RE	8.3E-10	218901_at	PLSCR4	9.4E-09
225760_at	MYSM1	8.9E-18	227350_at	HELLS	1.1E-07	201452_at	RHEB	8.3E-10	204979_s_at	SH3BGR	1.2E-08
227562_at	MAPKSP1	8.9E-18	214426_x_at	CHAF1A	1.2E-07	206516_at	AMH	8.4E-10	1554343_a_at	STAP1	1.2E-08
230029_x_at	UBR3	8.9E-18	223407_at	C16orf48	1.3E-07	1553030_a_at	SUOX	8.4E-10	219722_s_at	GDPD3	1.2E-08
225984_at	PRKAA1	1.0E-17	207746_at	POLQ	2.2E-07	204570_at	COX7A1	8.4E-10	210624_s_at	ILVBL	1.8E-08
1552347_at	CRYZL1	1.0E-17	219258_at	TIPIN	2.2E-07	213619_at	HNRNPH1	8.4E-10	211500_at	MAPK11	2.9E-08
230078_at	RAPGEF6	1.2E-17	38487_at	STAB1	3.2E-07	217178_at	RARG	8.7E-10	239602_at	---	3.3E-08
212982_at	ZDHHC17	1.2E-17	218911_at	YEATS4	3.2E-07	1557714_at	CTBP1	9.3E-10	221676_s_at	CORO1C	3.5E-08
225159_s_at	---	1.2E-17	208073_x_at	TTC3	3.3E-07	223567_at	SEMA6B	9.3E-10	210128_s_at	LTB4R	3.6E-08
202386_s_at	KIAA0430	1.2E-17	220085_at	HELLS	3.4E-07	230553_at	---	1.1E-09	210314_x_at	TNFSF13	3.6E-08
208073_x_at	TTC3	1.2E-17	219004_s_at	C21orf45	4.9E-07	204150_at	STAB1	1.1E-09	202856_s_at	SLC16A3	4.4E-08
223301_s_at	CCDC82	1.3E-17	204979_s_at	SH3BGR	5.9E-07	211296_x_at	UBC	1.1E-09	209499_x_at	TNFSF12-TNFSF13 /// TNFSF13	6.8E-08

212150_at	EFR3A	1.9E-17	215942_s_at	GTSE1	6.0E-07	222795_s_at	PLCXD1	1.1E-09	229756_at	---	8.4E-08
209898_x_at	ITSN2	2.0E-17	236969_at	---	6.1E-07	228684_at	ZNF503	1.3E-09	242579_at	BMPR1B	9.8E-08
239646_at	---	2.3E-17	208662_s_at	TTC3	6.2E-07	220080_at	FBXL8	1.4E-09	216388_s_at	LTB4R	1.0E-07
1557240_a_at	---	2.5E-17	214745_at	PLCH1	7.8E-07	209927_s_at	C1orf77	1.9E-09	211250_s_at	SH3BP2	1.3E-07
214917_at	PRKAA1	2.6E-17	204150_at	STAB1	7.8E-07	213559_s_at	ZNF467	2.0E-09	220030_at	STYK1	1.3E-07

Supplementary Table S5 continued

PIGP = 221689_s_at			TTC3 = 208662_s_at			VPS26C = 217309_s_at			DYRK1A = 209033_s_at		
probeset	Gene symbol	FDR	probeset	Gene symbol	FDR	probeset	Gene symbol	FDR	probeset	Gene symbol	FDR
202749_at	WRB	1.4E-26	208661_s_at	TTC3	2.6E-93	209399_at	HLCS	8.4E-17	202985_s_at	BAG5	1.4E-25
202325_s_at	ATP5J	3.2E-21	208073_x_at	TTC3	1.3E-72	232934_at	---	2.2E-11	226465_s_at	SON	9.8E-24
200818_at	ATP5O	8.8E-17	210645_s_at	TTC3	1.4E-70	203405_at	PSMG1	8.0E-11	230427_s_at	BAG5	2.6E-23
228239_at	FAM165B	3.5E-16	208663_s_at	TTC3	3.7E-52	221689_s_at	PIGP	8.0E-11	201800_s_at	OSBP	6.1E-22
228763_at	MDP-1	4.5E-16	208664_s_at	TTC3	2.0E-44	1554767_s_at	CRYZL1	2.7E-10	222543_at	DERL1	4.0E-21
203405_at	PSMG1	5.4E-16	1569472_s_at	TTC3	4.1E-37	207550_at	MPL	3.9E-10	225138_at	ZRANB1	4.1E-21
217927_at	SPCS1	6.3E-16	219767_s_at	CRYZL1	3.2E-24	203635_at	VPS26C	3.9E-10	217798_at	CNOT2	4.1E-21
219004_s_at	C21orf45	6.0E-15	226151_x_at	CRYZL1	1.2E-22	1557329_at	---	5.4E-10	224831_at	CPEB4	4.1E-21
219979_s_at	C11orf73	7.5E-15	218269_at	RNASEN	2.5E-22	222907_x_at	TMEM50B	1.3E-08	201880_at	ARIH1	4.1E-21
203550_s_at	C1orf2	1.0E-14	201085_s_at	SON	1.1E-21	204775_at	CHAF1B	1.5E-08	202097_at	NUP153	1.5E-20
218163_at	MCTS1	1.0E-14	1552347_at	CRYZL1	3.6E-21	1559679_a_at	---	1.7E-08	222182_s_at	CNOT2	1.5E-20
228283_at	CMC1	1.2E-14	218515_at	C21orf66	3.8E-21	212379_at	GART	5.4E-08	203250_at	RBM16	2.1E-20
213698_at	ZMYM6	1.4E-14	244813_at	---	3.0E-20	203920_at	NR1H3	5.5E-08	1556006_s_at	CSNK1A1	2.1E-20
210624_s_at	ILVBL	1.4E-14	243759_at	SFRS15	4.3E-19	219600_s_at	TMEM50B	5.7E-08	224905_at	WDR26	2.1E-20
203920_at	NR1H3	1.4E-14	1553227_s_at	BRWD1	1.1E-18	202749_at	WRB	6.2E-08	221230_s_at	ARID4B	3.1E-20
221599_at	C11orf67	1.4E-14	215596_s_at	RNF160	3.0E-18	200818_at	ATP5O	1.2E-07	222119_s_at	FBXO11	3.6E-20
218511_s_at	PNPO	4.7E-14	1553292_s_at	FLJ25006	1.2E-17	241742_at	PRAM1	1.2E-07	208866_at	CSNK1A1	3.7E-20

206790_s_at	NDUFB1	5.5E-14	242737_at	---	1.4E-17	212378_at	GART	1.4E-07	218172_s_at	DERL1	6.1E-20
228764_s_at	MDP-1	6.4E-14	201086_x_at	SON	8.0E-17	217445_s_at	GART	1.7E-07	1556007_s_ at	---	7.8E-20
219924_s_at	ZMYM6	6.5E-14	226465_s_at	SON	9.6E-17	38487_at	STAB1	2.1E-07	225268_at	KPNA4	7.8E-20
221600_s_at	C11orf67	8.6E-14	1554767_s_ at	CRYZL1	2.4E-16	226151_x_at	CRYZL1	2.4E-07	224898_at	WDR26	1.0E-19
222530_s_at	MKKS	1.1E-13	213000_at	MORC3	3.1E-16	207833_s_at	HLCS	3.2E-07	202582_s_at	RANBP9	1.0E-19
204979_s_at	SH3BGR	1.5E-13	214988_s_at	SON	7.3E-16	213715_s_at	KANK3	3.5E-07	206015_s_at	FOXJ3	1.2E-19
200642_at	SOD1	1.5E-13	239407_at	---	8.8E-16	204150_at	STAB1	3.7E-07	218107_at	WDR26	1.2E-19
229211_at	DUSP28	1.6E-13	215731_s_at	MPHOSPH9	1.9E-15	231538_at	C11orf1	4.6E-07	243463_s_at	RIT1	1.3E-19
209449_at	LSM2	2.0E-13	218953_s_at	PCYOX1L	2.1E-15	228239_at	FAM165B	6.1E-07	212293_at	HIPK1	1.5E-19
229190_at	---	2.3E-13	212996_s_at	URB1	4.5E-15	208395_s_at	URB1	6.6E-07	212997_s_at	TLK2	3.2E-19
223407_at	C16orf48	2.7E-13	207231_at	DZIP3	5.7E-15	1566501_at	---	9.7E-07	221749_at	YTHDF3	3.3E-19
225014_at	LOC389203	2.7E-13	225182_at	TMEM50B	7.9E-15	202855_s_at	SLC16A3	1.2E-06	218386_x_at	USP16	4.4E-19
218081_at	C20orf27	2.9E-13	219797_at	MGAT4A	8.5E-15	230302_at	---	2.3E-06	225694_at	CRKRS	7.2E-19
218773_s_at	MSRB2	2.9E-13	205250_s_at	CEP290	8.5E-15	212996_s_at	URB1	2.4E-06	212408_at	TOR1AIP1	9.3E-19
228597_at	C21orf45	4.7E-13	219482_at	SETD4	8.8E-15	202993_at	ILVBL	2.6E-06	204194_at	BACH1	1.2E-18
202993_at	ILVBL	5.1E-13	235698_at	ZFP90	8.8E-15	219767_s_at	CRYZL1	2.7E-06	225229_at	AFF4	1.3E-18
218154_at	GSDMD	5.3E-13	226848_at	---	9.5E-15	219924_s_at	ZMYM6	2.8E-06	213549_at	---	1.3E-18
228972_at	---	6.9E-13	219280_at	BRWD1	1.0E-14	232164_s_at	EPPK1	2.9E-06	221918_at	PCTK2	1.5E-18
223001_at	OSTC	7.6E-13	222310_at	SFRS15	1.4E-14	211163_s_at	TNFRSF10C	3.6E-06	224897_at	WDR26	1.5E-18
229671_s_at	C21orf45	9.1E-13	200944_s_at	HMGN1	1.6E-14	216388_s_at	LTB4R	5.1E-06	225284_at	DNAJC3	2.1E-18
228730_s_at	SCRN2	1.0E-12	201935_s_at	EIF4G3	1.9E-14	229623_at	FLJ12993	5.4E-06	226633_at	RAB8B	2.1E-18
204168_at	MGST2	1.0E-12	206572_x_at	ZNF85	2.0E-14	218123_at	C21orf59	5.4E-06	201636_at	FXR1	2.1E-18
202678_at	GTF2A2	1.0E-12	238119_at	---	2.2E-14	1556308_at	PRRT3	5.6E-06	203593_at	CD2AP	2.2E-18
223421_at	CYHR1	1.1E-12	231960_at	BRWD1	2.9E-14	224414_s_at	CARD6	5.6E-06	238719_at	---	2.3E-18
211370_s_at	MAP2K5	1.1E-12	225174_at	DNAJC10	3.2E-14	1554430_at	FAM165B	5.6E-06	1557675_at	RAF1	2.5E-18
230326_s_at	C11orf73	1.1E-12	213186_at	DZIP3	3.8E-14	238071_at	LCN10	5.9E-06	201845_s_at	RYBP	3.0E-18
202875_s_at	PBX2	1.1E-12	212980_at	USP34	3.8E-14	208664_s_at	TTC3	6.0E-06	213579_s_at	EP300	4.0E-18

226151_x_at	CRYZL1	1.7E-12	222244_s_at	TUG1	4.7E-14	1553030_a_at	SUOX	6.6E-06	227373_at	ATXN1L	4.0E-18
211747_s_at	LSM5	2.3E-12	222311_s_at	SFRS15	5.5E-14	208130_s_at	TBXAS1	6.8E-06	213128_s_at	UBE3A	4.0E-18
226728_at	SLC27A1	2.7E-12	212725_s_at	TUG1	6.1E-14	229698_at	---	7.4E-06	228822_s_at	USP16	4.0E-18
225003_at	TMEM205	3.8E-12	220175_s_at	CBWD1 /// CBWD2 /// CBWD3 /// CBWD5 /// CBWD6 /// CBWD7	6.7E-14	219004_s_at	C21orf45	7.4E-06	225267_at	KPNA4	4.0E-18
208818_s_at	COMT	3.9E-12	213677_s_at	PMS1	8.3E-14	235134_at	---	7.4E-06	204435_at	NUPL1	5.0E-18
219600_s_at	TMEM50B	4.2E-12	209298_s_at	ITSN1	8.6E-14	1552347_at	CRYZL1	8.8E-06	222990_at	UBQLN1	5.5E-18

NOTE: Probesets are ordered on FDR per probeset in the CRA, ascending. The top 50 genes that correlate to the selected probesets as described in the row above are shown.
Abbreviations: FDR, false discovery rate

Supplementary Figure S1

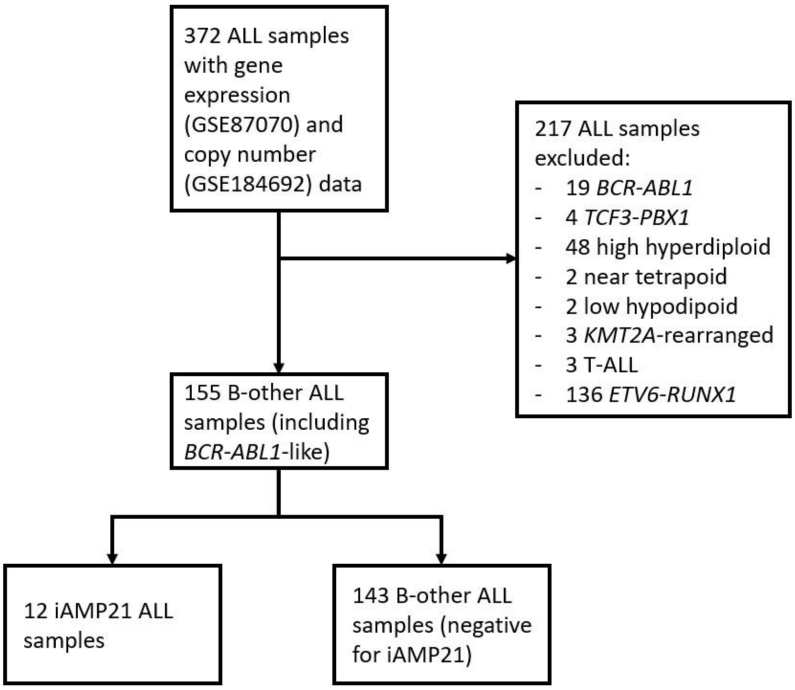

Supplementary Figure S1: Selection of the samples. Diagram showing the selection process of the 155 B-other samples.

Supplementary Figure S2:

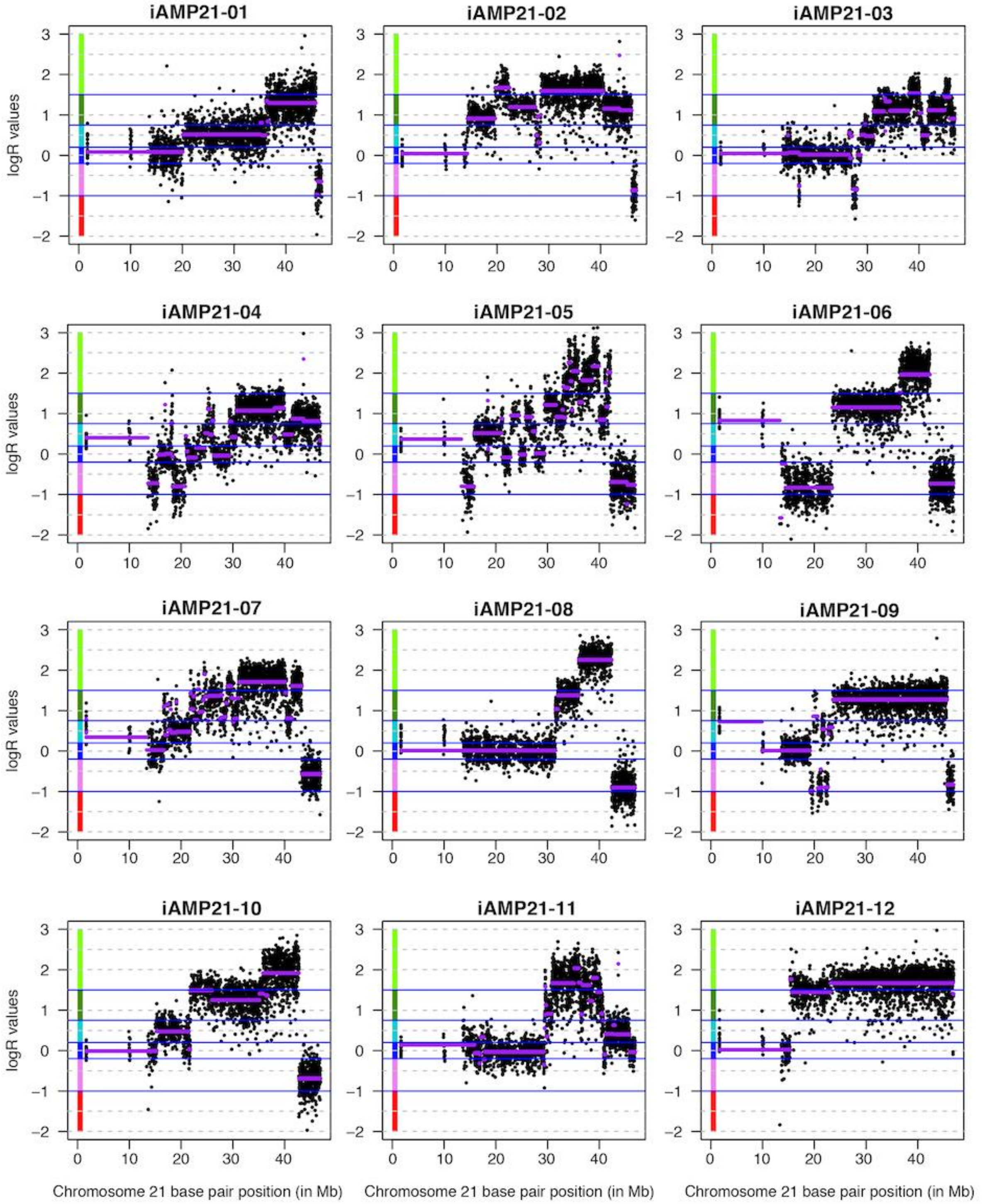

Supplementary Figure S2: Chromosome 21 copy number profile in all 12 iAMP21 patients. Copy number profile of chromosome 21 of all 12 iAMP21 patients identified in this study. Dots represent normalized logR values of the individual probes, purple line shows the segmented LogR values. Chromosome 21 base pair position is shown on the x-axis (reference genome hg18), with Log2 ratio value on the y-axis. Blue lines show the LogR value borders as used in figure 1A, colors on the y-axis represent the colors as shown in Figure 1A.

Supplementary Figure S3:

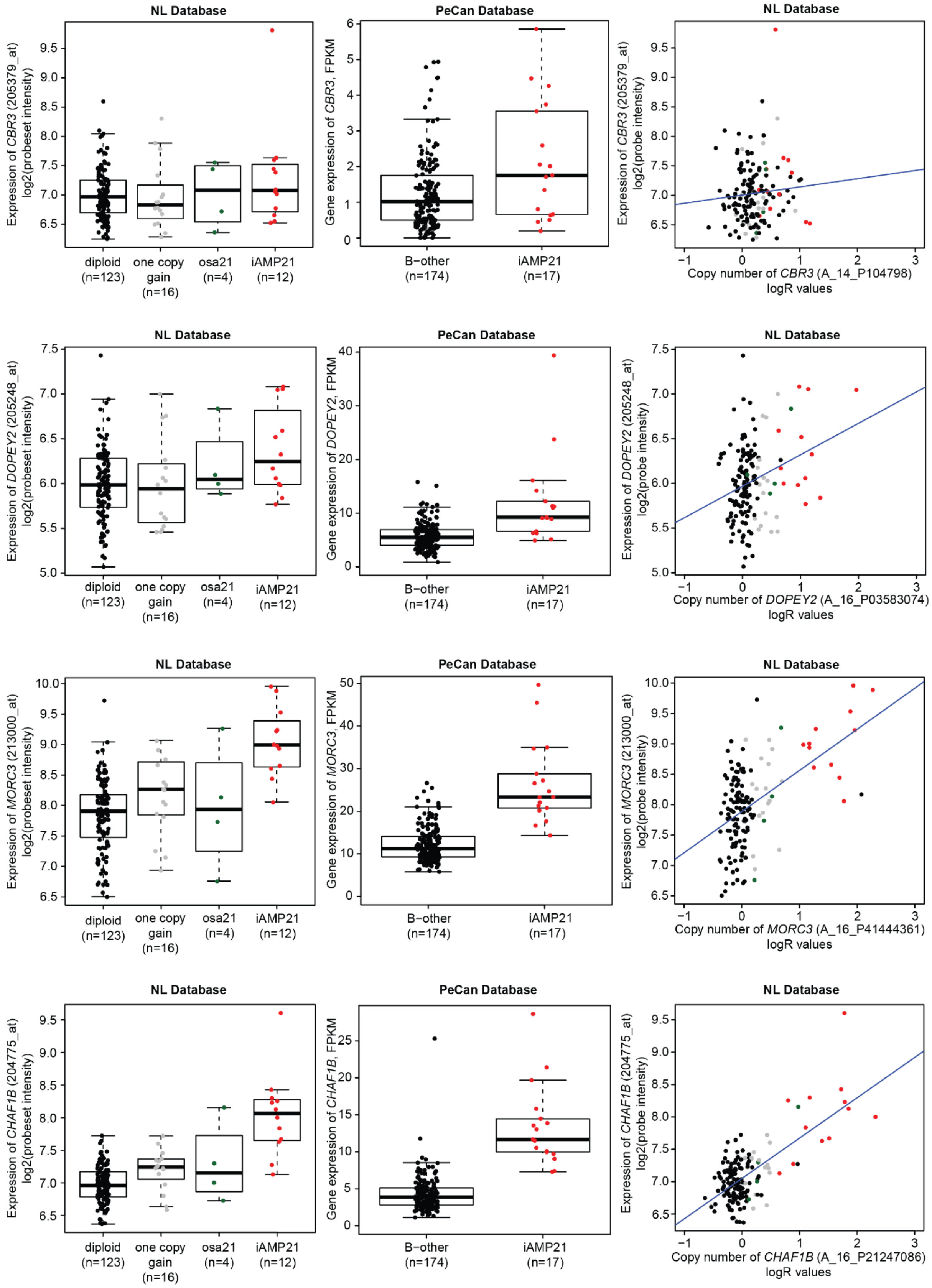

Supplementary Figure S3 continued

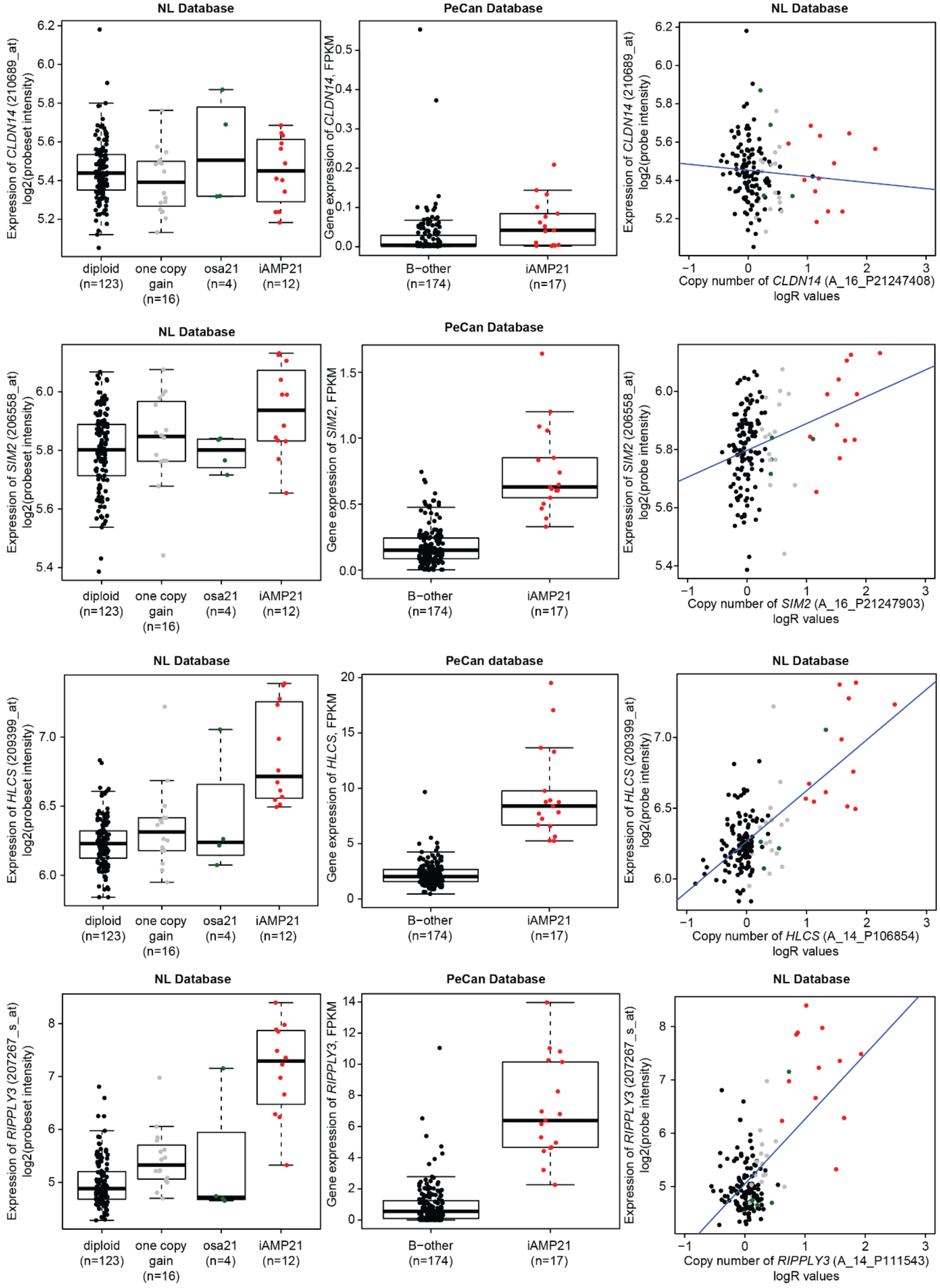

Supplementary Figure S3 continued

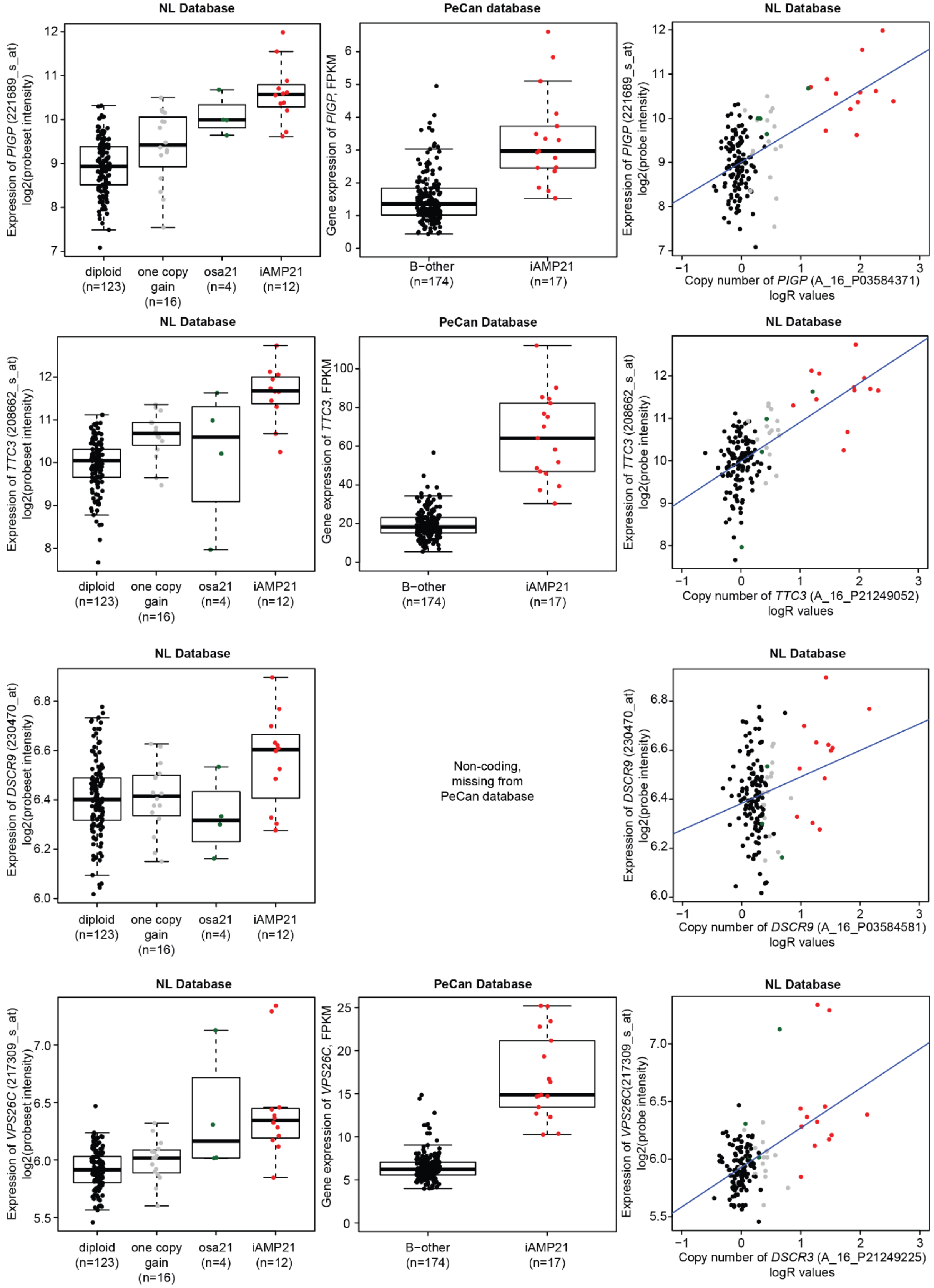

Supplementary Figure S3 continued

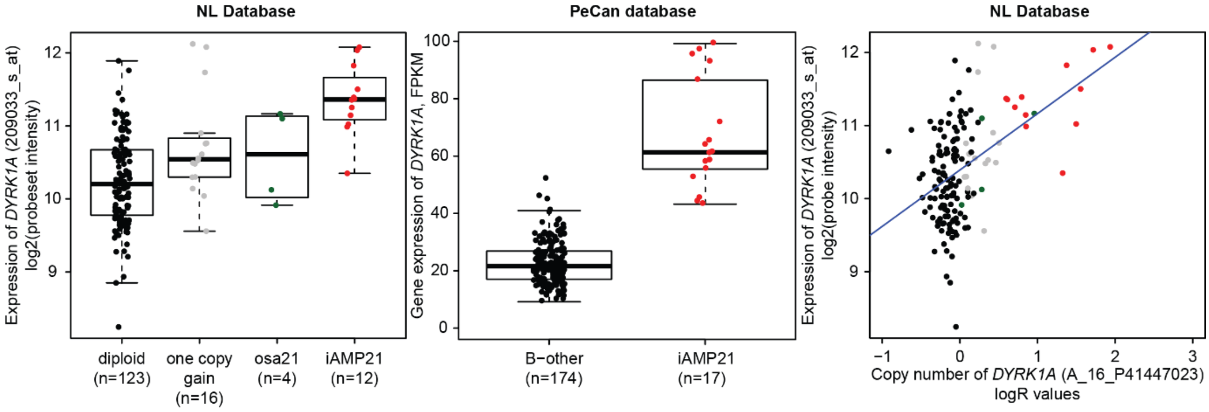

Supplementary Figure S3: Gene expression levels and correlation with copy number of all 13 CRA genes. Per gene, left panel shows the expression levels of the CRA gene (log2 intensities) categorized based on chromosome 21 copy number aberrations determined by aCGH: diploid, one copy gain, other structural aberration on chromosome 21 (osa21), and iAMP21. Middle panel shows expression of the CRA gene (FPKM) in the PeCan database. Selected patients are categorized based on iAMP21 status, as described in the PeCan database. Right panel shows the correlation between copy number (normalized logR values, x-axis) and gene expression (log2 values, y-axis). Patients are colored based on chromosome 21 copy number aberrations determined by aCGH: diploid, one copy gain, other structural aberration on chromosome 21 (osa21), and iAMP21. Blue line represents the linear regression trendline using all samples shown in this figure.

Supplementary Figure S4

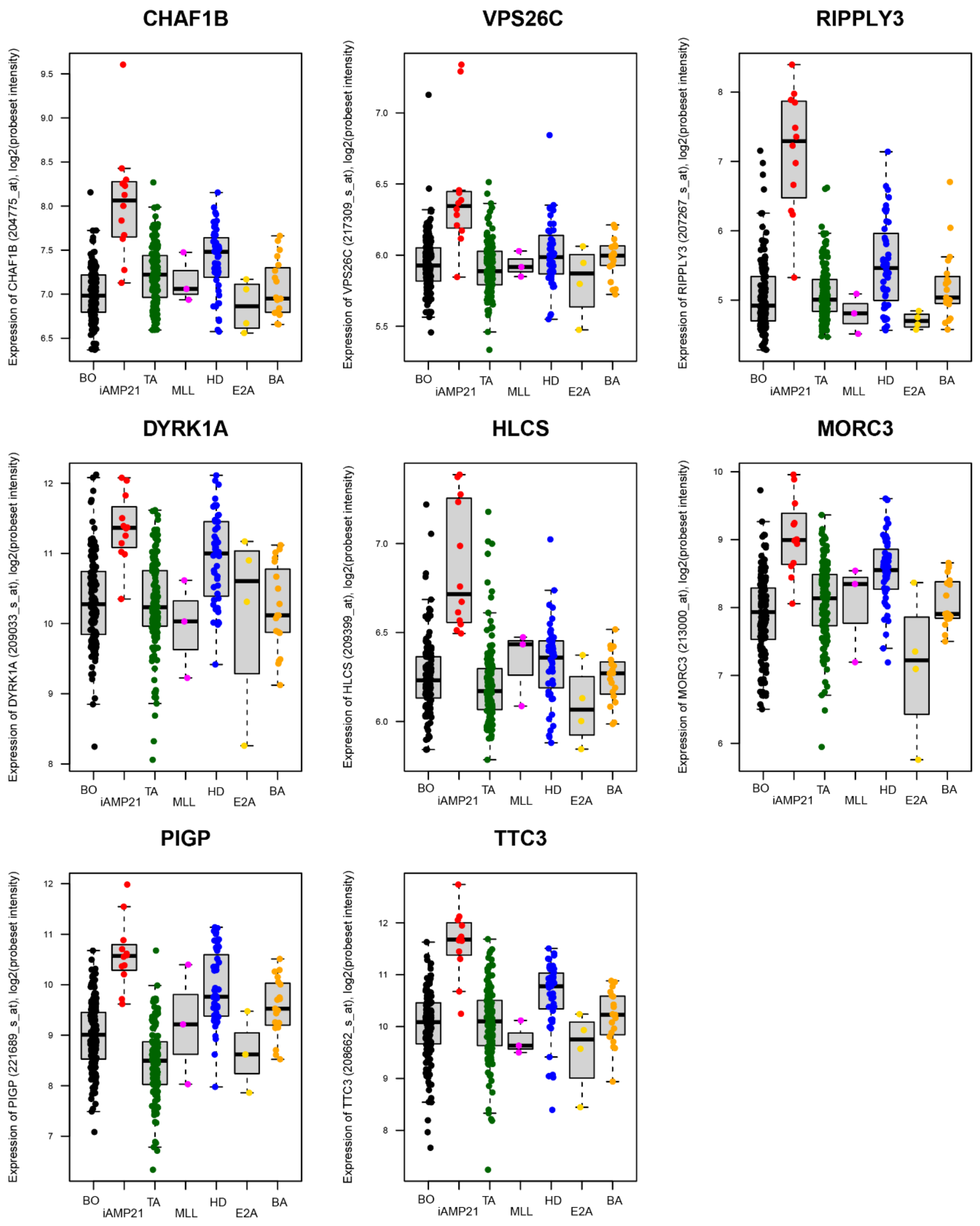

Supplementary Figure S4: Gene expression levels of the 8 selected CRA genes in all main subtypes. Per gene, panel shows the expression levels of the CRA gene (log2 intensities) categorized based on BCP-ALL subtype. Abbreviations: BO, B-other, iAMP21, intrachromosomal amplification of chromosome 21; TA, *ETV6-RUNX1*; MLL, *KMT2A*-rearranged; HD, high hyperdiploid; E2A, *TCF3-PBX1*; BA, *BCR-ABL1*.
